# Nonparametric Assessment of the Calibration of Individualized Treatment Effects

**DOI:** 10.1002/sim.70724

**Published:** 2026-08-25

**Authors:** Mohsen Sadatsafavi, Jeroen Hoogland, Thomas P. A. Debray, John Petkau

**Affiliations:** ^1^ Respiratory Evaluation Sciences Program, Collaboration for Outcomes Research and Evaluation, Faculty of Pharmaceutical Sciences The University of British Columbia Vancouver Canada; ^2^ Department of Epidemiology and Data Science Amsterdam University Medical Centers Amsterdam the Netherlands; ^3^ Smart Data Analysis and Statistics B.V. Utrecht Germany; ^4^ University Medical Center Göttingen Göttingen Germany; ^5^ Department of Statistics The University of British Columbia Vancouver Canada

**Keywords:** calibration, clinical prediction models, hypothesis testing, inference, treatment benefit

## Abstract

An important aspect of the performance of algorithms that predict individualized treatment effects (ITEs) is moderate calibration, that is, the average treatment effect among individuals with predicted treatment effect of z being equal to z. The assessment of moderate calibration is a challenging task on two fronts: counterfactual responses are unobserved, and quantifying the conditional response function for models that generate continuous predicted values requires regularization. Perhaps because of these challenges, there is currently no inferential method for the null hypothesis that an ITE model is moderately calibrated in a population. In this work, we propose nonparametric methods for the assessment of moderate calibration of ITE models for binary outcomes using data from a randomized trial. These methods simultaneously resolve both challenges, resulting in novel graphical, numerical, and inferential methods for the assessment of moderate calibration. The key idea is to formulate a stochastic process for the cumulative prediction errors that obeys a functional central limit theorem, enabling the use of the properties of Brownian motion for asymptotic inference. We propose two approaches to construct this process from a sample: a conditional approach that relies on predicted risks (often an auxiliary output of ITE models), and a marginal approach based on replacing the cumulative conditional expected value and variance terms with their marginal counterparts. Numerical simulations confirm the desirable properties of both approaches and their ability to detect miscalibration of different forms. We use a case study to provide suggestions on graphical presentation and the interpretation of results. Moderate calibration of predicted ITEs can be assessed without requiring regularization techniques or making assumptions about the functional form of treatment response. The accompanying cumulcalib R package implements this method ( https://cran.r‐project.org/package=cumulcalib).

## 1 Introduction

Due to their direct relevance to clinical decision‐making, prediction algorithms that produce quantitative estimates of individualized treatment effect (ITE) have been the subject of much recent development [1, 2, 3]. A common paradigm in medical practice is to offer treatment to patients at high risk of untoward clinical outcomes, where the risk is quantified via the application of prognostic markers or risk prediction models [4]. Yet, evidence that these patients actually benefit more than those with low outcome risk is often lacking. Indeed, this risk‐based approach toward treatment recommendation is often based on strong and untested assumptions such as a monotonic association between treatment effect and outcome risk. By directly producing an estimate of treatment effect given an individual's salient characteristics, ITE models enable more principled decision‐making. An example of such a model is the GRACE 3.0 ITE predictor for early invasive management of non‐ST‐elevation acute coronary syndrome [5], developed using data from the VERDICT trial. Another example is the ITE model for the effect of oxygen‐saturation targets on mortality in patient under intensive care, based on data from the PILOT trial [6].

Before being implemented for patient care in a population, the performance of an ITE model should be assessed using an independent sample from that population—a practice commonly referred to as (external) validation [7, 8]. A critical aspect of ITE model performance in a validation study is calibration: the ability of the model to generate predictions that are close to their true counterparts [9]. Poorly calibrated ITE models may yield individualized predictions that are exaggerated or do not adequately reflect actual benefit. Consequently, they can result in misguided clinical decisions by care providers and may mislead patients in making treatment choices that are not aligned with their preferences.

In the context of risk prediction models, calibration is defined at different levels [10, 11, 12]. The most basic form is calibration in the large (a.k.a. mean calibration), which refers to the average predicted and average observed risks being equal. Of more interest is assessing whether average observed risk among all individuals with a given predicted risk is equal to that predicted risk. This form of calibration is interchangeably referred to as “weak” (e.g., by Pepe et al. [10] and Blanche et al. [12]) or “moderate” (e.g., by van Calster et al. [11]) calibration. Henceforth we use moderate calibration for consistency with our directly‐related previous work [13]. This terminology is to contrast it with “strong” calibration, which refers to the average observed and predicted risks being equal within every subgroup defined by a combination of predictor values [10, 11, 12]. However, it is argued that strong calibration is neither achievable nor is it strictly desirable [11]. This makes mean calibration (often the very first step in calibration assessment) and moderate calibration of particular relevance. In practice, mean calibration is assessed by comparing the average observed and predicted risks, while moderate calibration is often assessed visually using a flexible calibration plot, which depicts the conditional mean of observed risk as a function of predicted risk. When predicted risks are continuous with no or few ties, estimating this conditional mean requires some form of smoothing or grouping. Scalar metrics have also been proposed to summarize it [14, 15, 16]. Because these visualizations and summary metrics rest on an estimated conditional‐mean function, their computation depends on tuning parameters (e.g., the number of bins, or the LOESS bandwidth and polynomial degree), and the choice of such parameters can have a noticeable impact on the assessment.

Principles of calibration hierarchy can largely be extended to ITE models [9]. Mean calibration can be examined by comparing the observed average treatment effect (ATE) and the average of predicted effects. For weak/moderate calibration, van Klaveren et al. [17] quantified the difference between predicted and observed absolute treatment effects in quartiles of predicted effects. Maas et al. [18] proposed applying local smoothing functions to match‐based estimates of conditional treatment effect, producing graphical presentations and numerical metrics for the assessment of moderate calibration. Hoogland et al. [8] proposed fitting logit models for outcome risk separately within control and treatment groups, from which the calibration function for ITEs can be derived. However, these approaches require grouping of data into bins, applying local smoothing methods, or using parametric regression modeling.

Calibration can also be approached as a formal inference problem. Inference on mean calibration for a risk prediction model entails testing whether the average predicted risk equals the average observed risk. Traditionally, inference on moderate calibration for risk prediction models has been based on the Hosmer–Lemeshow test, albeit this test is criticized for being sensitive to the inevitably arbitrary number of groups (often set to 10) [19]. For ITE models, mean calibration can be tested using standard methods (e.g., testing for the equality of the average observed and predicted treatment effect). However, to the best of our knowledge, there are currently no inferential methods available for testing whether an ITE model is moderately calibrated. This is an important methodological gap, as achieving moderate calibration is an important criterion for many clinical applications to proceed with the implementation of the model or assessment of impact in further studies. Miscalibrated models might result in loss of clinical benefit, as a treatment decision based on the predicted ITE might be different from the decision had the true ITE been known.

Recently, nonparametric approaches have been proposed for assessing moderate calibration of risk prediction models based on the behavior of cumulative prediction errors that do not require smoothing or grouping of observations [13, 20, 21]. The core idea is that if the model is calibrated, the cumulative sum of prediction errors should stay near zero, whereas for miscalibrated models, the cumulative errors will tend to drift away from zero along their path. These approaches yield a new class of visualizations, summary metrics, and inferential tools for moderate calibration, without requiring regularization. In particular, they enable formal inference on the null hypothesis that “this model is moderately calibrated in this population,” based on asymptotic tests that rely on known properties of Brownian motion. It would be appealing to extend this line of work to the assessment of calibration of ITE models. This extension is challenging, as, unlike risks, treatment benefits are not typically observable at the individual level.

The purpose of this work is to propose non‐parametric, tuning‐parameter‐free approaches for the assessment of moderate calibration of ITE models. Because of their causal underpinnings, ITE models are often trained using data from randomized controlled trials (RCTs). As such, we focus on validation studies based on RCT data, and, as a first step, we consider binary outcomes. The rest of this manuscript is structured as follows: after outlining the context, we briefly review nonparametric calibration assessment for predicted risks. We then extend this idea to ITE models by creating a stochastic process that converges asymptotically to standard Brownian motion, which is the basis for two approaches for constructing this process in the sample. We conduct simulation studies examining the performance of tests based on both approaches. These developments are exemplified in a case study where we showcase the assessment of moderate calibration on the cumulative domain. We conclude by discussing how these methods can be added to the toolbox for evaluating ITEs and suggest avenues for future research, including their use in non‐randomized settings.

## 2 Methods

### 2.1 Notation and Context

We focus on binary outcomes (e.g., in‐hospital mortality due to a severe COVID‐19 infection), and assume the outcome is an untoward event, and, as such, treatment effect is in terms of absolute risk reduction. For treatments that increase the probability of a desirable outcome, all subsequent developments are applicable after relabeling the treatment and control interventions. Let $Y^{\left(0\right)}$ and $Y^{\left(1\right)}$ be the potential outcomes under control and treatment, respectively, both taking a value in {0,1}. Let Y be the observed response, taking a value in {0,1}. The treatment assignment indicator is a (0: control, 1: treatment).

An ITE model is a deterministic function that for a given patient maps their covariate pattern to a predicted treatment effect δ, taking a value in (−1,1), which is claimed to be a good approximation for $E\left(Y^{\left(0\right)}-Y^{\left(1\right)}\right)$ among all individuals with the same covariate pattern. The ITE model can be a regression‐based or machine‐learning algorithm (as the functional form of the mapping algorithm is not of direct relevance). Many ITE models are based on risk prediction models that separately make predictions for outcome risk under each treatment scenario, either from separate models (e.g., a T learner) or a model with treatment as a predictor (a S learner) [22]. These models therefore yield counterfactual predictions: the expected outcome if treated and the expected outcome if receiving the control treatment among all individuals with the same covariate patterns. The predicted ITE is the difference between these predicted values. For such models, by π we refer to the “baseline” risks, which are claimed to be a good approximation for $E\left(Y^{\left(0\right)}\right)$. However, some modeling approaches can generate predicted ITEs without modeling the underlying risks [23, 24].

For our validation task, we assume we have access to an iid sample of size n from a parallel‐arm RCT comparing the treatment and control of interest. For the ith individual, we have access to the following items: $a_{i}$, representing treatment assignment, the binary response variable $Y_{i}$, and the predicted ITE $\delta_{i}$. For ITE models that also generate predicted risks, we will also have access to the predicted baseline risk $\pi_{i}$ (which takes a value in (0,1)). We consider δs and πs (if the ITE model also produces them) fixed at their calculated values. While not strictly necessary, for ease of derivations, we assume no ties in predicted benefits (i.e., at least one of the predictors is a continuous variable).

Our goal is to construct a stochastic process {S}, that is, a process with an implied time and an implied location component, based on the cumulative sum of prediction errors that asymptotically converges to Brownian motion, W(t), on the [0,1] time interval, if the ITE model is moderately calibrated. This enables us to extend the previously developed nonparametric approaches for calibration assessment of risk prediction models to ITE models.

### 2.2 Nonparametric Approaches for Moderate Calibration of Predicted Risks

In this section, we briefly review nonparametric methods for the assessment of moderate calibration of algorithms that predict outcome risks given an individual's predictor pattern. These methods form the basis of our subsequent developments for ITE models. To avoid introducing new notation, in this section we assume none of the individuals has received the treatment. As such, the baseline predicted risks $\pi =\left(\pi_{1},\pi_{2},\ldots ,\pi_{n}\right)$ remain pertinent predicted risks for the observed responses. For such predicted risks, the null hypothesis that the model is moderately calibrated is defined as ∀z:E(Y|π=z)=z. If a model generates continuous predicted risks, estimating the conditional mean E(Y|π) requires regularization (binning or smoothing). The core idea of nonparametric assessment is that one can instead examine moderate calibration on the cumulative sum domain.

We start by indexing observations after ordering them from the small to large values of the predicted risks πs. We then construct the scaled cumulative sums of the prediction errors. For the kth observation, this is

(1) $$C_{k}=\frac{1}{n}\sum_{i=1}^{k}\left(Y_{i}-\pi_{i}\right),\text{for}\;k=1,2,\ldots ,n.$$

It follows immediately from the definition of moderate calibration that $\forall k:E\left(C_{k}\right)=0$ under the null hypothesis. This expectation can be estimated from the data, without any need for regularization.

If the model is not moderately calibrated, the $C_{k}$s tend to deviate substantially from zero along their path. Tygert proposed plotting $C_{k}$ as a function of k/n to inspect such deviation [20]. The degree of this deviation can be summarized into scalar metrics. One such metric, proposed by Arrieta‐Ibarra et al. [21], is the scaled maximum absolute value of cumulative prediction errors:

(2) $$C^{*}=\max_{k}|C_{k}|.$$

This metric summarizes the degree of miscalibration, and can be interpreted as the maximum absolute cumulative prediction error, as an alternative to scalar summary metrics of miscalibration such as $E_{\max}$ and the integrated calibration index (ICI) [14, 16]—one whose computation does not involve any tuning parameters (e.g., LOESS bandwidth) and thus results in an objective, reproducible summary of miscalibration.

Importantly, Arrieta‐Ibarra et al. argued that under the null hypothesis that the risk prediction model is calibrated, the distribution of a properly scaled version of $C^{*}$ converges, as n→∞, to the distribution of the maximum absolute deviation of Brownian motion: $nC^{*}/\sqrt{\sum_{i=1}^{n}\pi_{i}\left(1-\pi_{i}\right)}\overset{d}{\to }\sup_{t\in \left[0,1\right]}|W\left(t\right)|$. This is the test statistic of their proposed asymptotic test for moderate calibration (henceforth referred to as the Brownian Motion [BM] test).

Neither Tygert nor Arrieta‐Ibarra et al. explicitly discussed constructing a stochastic process—a process with a time and a location component—for prediction errors. The implied process in $C_{k}$, with time increments of 1/n and location changes of (Y−π)/n, is heteroscedastic, as the variances π(1−π) are variable but the time increments are all equal. As such, while certain summaries of this process converge to their counterparts for W(t) under $H_{0}$, this implied process does not achieve point‐wise convergence to W(t).

Sadatsafavi and Petkau [13] proposed turning these cumulative sums into a stochastic process that converges at all points to W(t), thus opening the door for taking full advantage of the properties of the latter. Let $s_{k}^{2}=\sum_{i=1}^{k}\pi_{i}\left(1-\pi_{i}\right)$ be the cumulative sum of variances up to the kth observation. They defined the following standardized process:

(3) $$\left\{S\right\}=\left\{\begin{array}{ll} t_{k}=s_{k}^{2}/s_{n}^{2} & \left(\text{time values}\right)\\ S_{k}=\frac{nC_{k}}{s_{n}} & \left(\text{location values}\right) \end{array}\right.$$

(with $t_{0}=0$ and $S_{0}=0$). The time increments are scaled to sum to 1, and the variances of the location changes are scaled to be equal to the time increments. This process is, under $H_{0}$, a martingale with independent and strictly bounded increments, with $s_{n}^{2}\to \infty$ as n→∞. Thus, Brown's martingale CLT establishes that the continuous process based on linear interpolation of successive ($t_{k},S_{k}$) points converges weakly to W(t) on the [0,1] time interval [25].

As an immediate application of this convergence, they proposed a two‐part “bridge” test. This test is motivated by the following observations. First, under $H_{0}$, $S_{n}\overset{d}{\to }W\left(1\right)∼\text{Normal}\left(0,1\right)$. Thus, $S_{n}$, the test statistic for the first part (based on the terminal position of the random walk), is a *z*‐score for mean calibration, a necessary condition for moderate calibration. Second, the path of a “bridged” Brownian motion, which is defined as the difference between W(t) and the line that connects its endpoints (also known as the Brownian bridge [26]) is independent of its terminal value: ∀t∈[0,1):W(t)−tW(1)⊥W(1). As miscalibrated models tend to generate large deviations from this path, examining the maximum absolute deviation of the bridged random walk provides an opportunity, independently of $S_{n}$, for assessing moderate calibration. The test statistic for the second part is thus $S^{*}=\max_{k}\left(\;|S_{k}-t_{k}S_{n}|\;\right)$, with $S^{*}\overset{d}{\to }\sup_{t\in \left[0,1\right]}|W\left(t\right)-\mathrm{tW}\left(1\right)|$ under $H_{0}$. The distribution of the maximum absolute deviation of the Brownian bridge is the Kolmogorov distribution, the asymptotic null distribution of the two‐sided one‐sample Kolmogorov–Smirnov test statistic [27, 28]. The *p* values of each part are combined via Fisher's method to generate a unified *p*‐value for moderate calibration. Simulations confirmed the intuition that, being based on two independent statistics, the bridge test generally has higher power than the BM test. The authors also proposed graphical visualization of the homoscedastic {S} process as an alternative to the presentation of the heteroscedastic scaled cumulative calibration errors (C).

**FIGURE 1:**
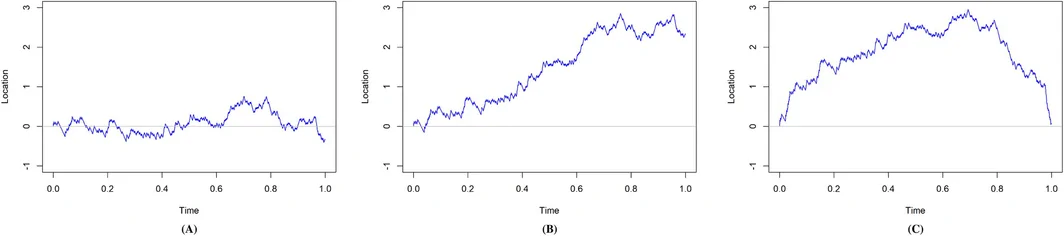
Example of the {S} process for a calibrated model* (A), a model that underestimates the actual outcome (B), and an overfitted model that makes extreme predictions (C). *The construction and interpretation of the S process are the same for risk‐prediction and ITE models: The qualitative reading of the trajectory as random fluctuation (A), systematic drift (B), or an inverse‐U departure (C), carries over directly. The true model is logit(P(Y=1))=−1+X with X∼Normal(0,1). The three prediction models are as follows: (A) Same as the true model; (B) logit(P(Y=1))=−1.2+X; (C) logit(P(Y=1))=−1.2+1.2X. Results are from a random dataset of 1000 observations. Time and Location axes refer to their corresponding elements in Equation (3). Note the random variation of cumulative errors in Panel A (typical of a calibrated model), the systematic increase in Panel B (indicating a systematic underestimation of risks), and the inverse U‐shape pattern in Panel C (indicating an over‐dispersed model that makes overly extreme predictions—underestimating risks when they are low and overestimating them when they are high).

Figure 1 provides examples of the visualization of the {S} process and typical miscalibration patterns. While this exemplary process is built around a risk prediction model, the interpretation of the process will be identical for ITE models.

### 2.3 Nonparametric Approaches Toward Calibration of ITE Models

Our main purpose is to generalize the above‐mentioned developments to the assessment of the calibration of ITE models. Before proceeding to main developments, we note that for ITE models that predict risks under control and treatment, predicted ITEs are the difference between these predicted risks. If predicted risks under both treatment conditions are to be communicated to the patient and be used in decision‐making, it is natural to demand that they both be moderately calibrated [8]. For this purpose, one can define a compound null hypothesis as $H_{0A}:\forall z:E\left(Y^{\left(0\right)}|\pi =z\right)=z$ and $H_{0B}:\forall z:E\left(Y^{\left(1\right)}|\pi =z\right)=z-\delta$. In the context of a randomized trial, and under classical assumptions for the potential outcomes framework (consistency, exchangeability, positivity, and no interference [29]), these hypotheses can also be written as $H_{0A}:\forall z:E\left(Y|a=0,\pi =z\right)=z$ and $H_{0B}:\forall z:E\left(Y|a=1,\pi =z\right)=z-\delta$. Because the subgroups of treated and control individuals are independent of each other, this compound null hypothesis can be tested via separate nonparametric assessment of the calibration of predicted risks within each treatment group. A unified *p*‐value can be generated via Fisher's method. A visualization of this approach would involve plotting the {S} processes within each treatment group. However, this approach fails to focus the attention on the component of interest here which is ITE calibration. Indeed, the ITEs can remain calibrated despite miscalibrated predicted risks. The visualization of cumulative predicted risk errors also does not provide an opportunity for a direct assessment of the cumulative prediction errors for ITEs. Further, this approach is not applicable to ITE models that do not output predicted risks. As such, for the rest of this work we study stochastic processes that focus on the calibration of predicted ITEs.

In this context, the null hypothesis that an ITE model is moderately calibrated can be formulated as follows:

(4) $$H_{0}:\forall z:E\left(Y|a=0,\delta =z\right)-E\left(Y|a=1,\delta =z\right)=z.$$

#### 2.3.1 A Stochastic Process for Cumulative ITEs

Our goal is to create a standardized {S} process for cumulative ITEs with weak convergence to W(t), enabling the use of the above‐mentioned visualizations, summary indices, and inference for the assessment of ITE models.

To transform the assessment domain to cumulative prediction errors, we start by indexing the observations after ordering the sample from small to large values of predicted ITEs, such that $\forall i<j:\delta_{i}<\delta_{j}$. Had we access to both potential responses at the individual level, we would be able to study, similarly to the approach used for predicted risks, an appropriately standardized version of cumulative prediction errors $\sum_{i=1}^{k}\left(\left(Y_{i}^{\left(0\right)}-Y_{i}^{\left(1\right)}\right)-\delta_{i}\right)$. However, one of $Y^{\left(0\right)}$ and $Y^{\left(1\right)}$ is counterfactual and is therefore unobserved.

Our core idea is to work with the following estimator in lieu of observed cumulative treatment effects:

(5) $$\begin{array}{cc} & B_{k}=k\times \left[\text{average risk reduction}\;\mathrm{due}\;\text{to treatment in the first}\;k\;\text{subjects}\right]=\\ & k\left(\frac{\sum_{i=1}^{k}\left(1-a_{i}\right)Y_{i}}{\sum_{i=1}^{k}\left(1-a_{i}\right)}-\frac{\sum_{i=1}^{k}a_{i}Y_{i}}{\sum_{i=1}^{k}a_{i}}\right), \end{array}$$

for k=1,2,3,…,n. By convention we define 0/0=0. This process constructs an estimator of cumulative ITEs as k times the estimate of the ATE among the first k subjects.

Based on this estimator, we construct a martingale to which a functional CLT can be applied. To this end, for the kth observation, the conditional expected values and conditional variances of the increments given the history of the cumulative sum up to the kth observation are required [30]. Let $F_{k}$ denote all the information embedded in the history up to and including the kth observation. The increments (differences) $D_{k}$ and their conditional expectations ($\mu_{k}$) are defined as (we set $B_{0}=0$):

(6) $$D_{k}=B_{k}-B_{k-1},\;\;\mu_{k}=E\left(B_{k}-B_{k-1}|F_{k-1}\right).$$

The equations for $D_{k}$ and $\mu_{k}$ are provided in Appendix 1. Of particular interest are the centered increments:

(7) $$D_{k}-\mu_{k}=k\left[\left(1-a_{k}\right)\frac{Y_{k}-\pi_{k}^{*}}{\sum_{1}^{k}\left(1-a_{i}\right)}-a_{k}\frac{Y_{k}-\pi_{k}^{*}+\delta_{k}^{*}}{\sum_{i=1}^{k}a_{i}}\right],$$

where $\pi_{k}^{*}=E\left(Y|\delta =\delta_{k},a=0\right)$ and $\delta_{k}^{*}=E\left(Y|\delta =\delta_{k},a=0\right)-E\left(Y|\delta =\delta_{k},a=1\right)$ are, respectively, the expected baseline risk and expected ITE among individuals with the predicted ITE equal to $\delta_{k}$.

The scaled cumulative sum of the centered increments thus becomes:

(8) $$C_{k}=\frac{1}{n}\sum_{i=1}^{k}\left(D_{i}-\mu_{i}\right).$$

To create the {S} process for the application of Brown's martingale CLT [25], we need to standardize the time differences according to the cumulative conditional (on the history) variances. The individual conditional variances are

(9) $$\sigma_{k}^{2}=\mathrm{Var}\left(D_{k}|F_{k-1}\right)=k^{2}\left[\left(1-a_{k}\right)\frac{\pi_{k}^{*}\left(1-\pi_{k}^{*}\right)}{{\left(\sum_{i=1}^{k}\left(1-a_{i}\right)\right)}^{2}}+a_{k}\frac{\left(\pi_{k}^{*}-\delta_{k}^{*}\right)\left(1-\pi_{k}^{*}+\delta_{k}^{*}\right)}{{\left(\sum_{i=1}^{k}a_{i}\right)}^{2}}\right],$$

and the total conditional variance at point k is

(10) $$s_{k}^{2}=\sum_{i=1}^{k}\sigma_{i}^{2}.$$

Putting all these together, we construct the stochastic process of interest ({S}) as in Equation (3), with a time component of $t_{k}=s_{k}^{2}/s_{n}^{2}$ and a location component of $S_{k}=\frac{nC_{k}}{s_{n}}$.

Similar to the {S} process for risks, time steps in this process sum to one, and the conditional variances of the increments are scaled to be equal to the time increments. We note that the increments in this process ($D_{k}-\mu_{k}$) are independent, but unlike their counterparts for risks, are not strictly bounded—as $k/\sum_{i=1}^{k}a_{i}$ and $k/\sum_{i=1}^{k}\left(1-a_{i}\right)$ can take arbitrarily large values. The application of the functional CLT is no longer straightforward. However, in Appendix 2, we show that under general regularity conditions for parallel‐armed RCTs, this process does satisfy the Lindeberg condition for martingales, and Brown's martingale CLT establishes that the continuous process based on linearly interpolating successive points of {S} converges weakly to W(t) on the [0,1] time interval.

The conditional moments of the above process depend on calibrated ITEs and baseline risks. Indeed, under $H_{0}$, our claim is that $\delta =\delta^{*}$, and so we replace $\delta^{*}$ with δ in Equations (7) and (9). But a practical challenge remains which is the need for calibrated baseline risks $\pi^{*}$. Two approaches are developed to address this issue. The first uses predicted risks in place of $\pi^{*}$. The second replaces cumulative conditional moments of the above process with their marginal counterparts, which do not require knowing $\pi^{*}$ risks.

#### 2.3.2 Approach 1: A Conditional (On Predicted Risks) Process

For ITE models that produce predicted risks, an obvious solution is to replace $\pi^{*}$s with their predicted counterparts. This results in an approach that is conditional on the assumption that the predicted baseline risks are well‐calibrated within strata of predicted ITEs. One can then generate, just like for risk, the graph of $C_{k}$ as a function of k/n. As well, $C_{n}$ is a measure of mean calibration, albeit not the conventional one based on comparing the observed and predicted ATE ($B_{n}/n-\sum_{i=1}^{n}\delta_{i}/n$). Further, similarly as for risks, $C^{*}=\max_{k}|C_{k}|$ is a scalar metric that captures the degree of miscalibration: miscalibrated ITE models tend to generate larger $C^{*}$ values compared with calibrated ones.

The main application of this approach arises in models that construct predicted ITEs from predicted risks for treatment and control, where the calibration of predicted risks is first evaluated, and the risk prediction component is revised if necessary to ensure calibration. A related example is when a previously validated risk prediction model is combined with an estimated treatment effect from a clinical trial to generate ITE predictions. In such cases, it is reasonable to condition inference about the ITEs on the premise that the predicted risks are calibrated. However, this approach does not apply to ITE models that do not generate predicted risks.

#### 2.3.3 Approach 2: An Approximate Marginal (Independent of Predicted Risks) Process

If baseline risks are not available or cannot be trusted as being calibrated, it will not be possible to construct a martingale whose asymptotic behavior can be fully specified. This is because the conditional expected values ($\mu_{k}$) and variances ($s_{k}^{2}$) of the increments depend on baseline risks. However, one can approximate the cumulative sum of conditional expected values and variances using their marginal counterparts, whose values can be obtained without knowledge of the baseline risks.

Under $H_{0}$, as we claim $\delta =\delta^{*}$, at point k the expected number of avoided outcomes is $\sum_{i=1}^{k}\delta_{i}$; therefore, the expected value of $B_{k}-B_{k-1}$ can be approximated by ${\tilde{\mu }}_{k}=\delta_{k}$, and thus we approximate the scaled cumulative prediction errors as:

(11) $${\tilde{C}}_{k}=\frac{1}{n}\left(B_{k}-\sum_{i=1}^{k}\delta_{i}\right).$$

As well, we replace the cumulative sum of conditional variances up to point k with the total marginal variance of B at this point:

(12) $${\tilde{s}}_{k}^{2}=\mathrm{Var}\left(B_{k}\right)=k^{2}\left(\frac{p_{0}\left(1-p_{0}\right)}{\sum_{i=1}^{k}\left(1-a_{i}\right)}+\frac{p_{1}\left(1-p_{1}\right)}{\sum_{i=1}^{k}a_{i}}\right),$$

where $p_{0}=\frac{\sum_{i=1}^{k}\left(1-a_{i}\right)Y_{i}}{\sum_{i=1}^{k}\left(1-a_{i}\right)}$, $p_{1}=\frac{\sum_{i=1}^{k}a_{i}Y_{i}}{\sum_{i=1}^{k}a_{i}}$ are the sample estimates, up to the kth observation, of the outcome risk in each arm. The {S} process is defined based on these $\tilde{C}$ and ${\tilde{s}}^{2}$ values replacing their counterparts in Equation (3).

**FIGURE 2:**
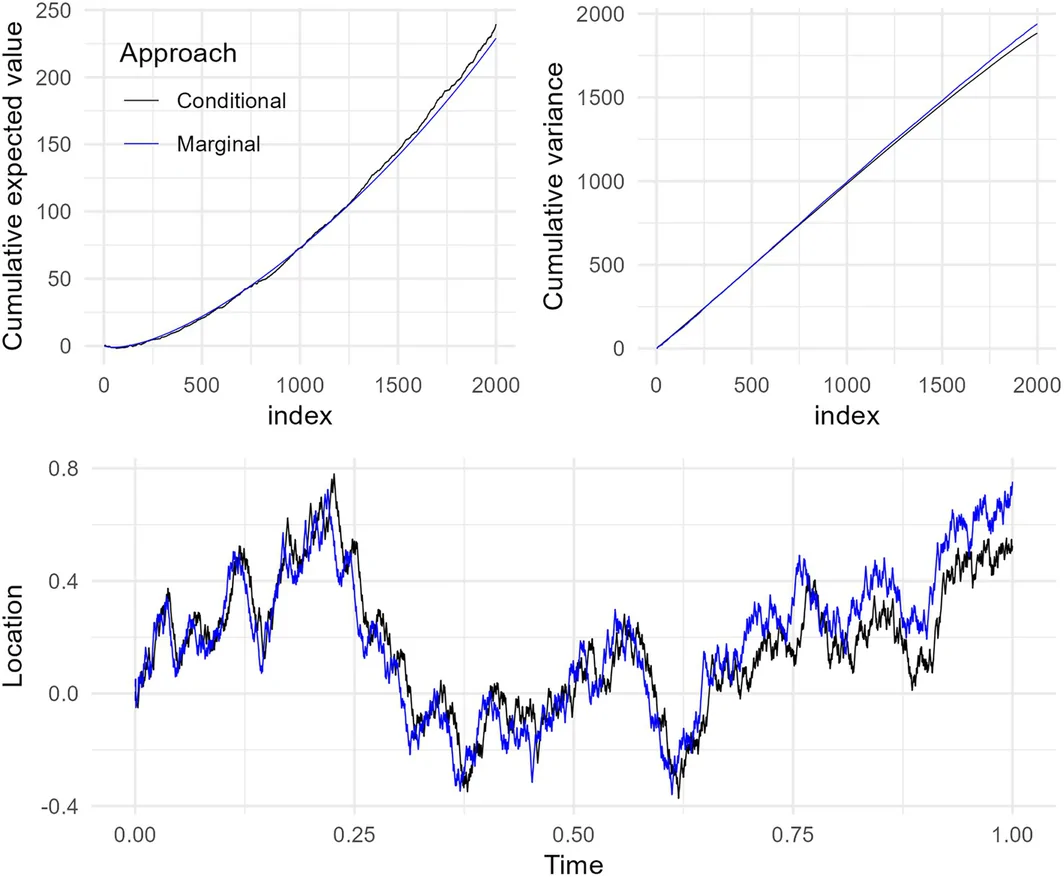
Cumulative conditional versus marginal expected values (top‐left), cumulative conditional versus marginal variances (top‐right), and corresponding {S} processes. Black: Conditional process; Blue: Marginal process. These are from an ITE model of the form $\text{logit}\left(\pi_{a}\left(x\right)\right)=0+0.25x+-0.5a+0.25\mathrm{xa}$ with x being the single predictor with a standard Normal distribution, and a simulated sample of size 2000.

We note that the asymptotic behavior of this marginal process is not fully supported by theory, and this approach remains heuristic in character. But our intuition is supported by the proximity of the resulting stochastic processes, and is confirmed in a more substantial fashion by the simulation studies of the next section. A visual example is provided in Figure 2. The top panels juxtapose the cumulative conditional and marginal moments from an exemplary sample. The bottom panel demonstrates the corresponding {S} processes. The general features of the processes are similar but shifted slightly both horizontally, due to discrepancies between variance terms, and vertically, due to discrepancies between expected values. We emphasize that this marginal process only approximates the stochastic process of interest. Specifically, while the marginal variances generally tend to increase, we might occasionally see ${\tilde{s}}_{k}^{2}<{\tilde{s}}_{k-1}^{2}$, resulting in occasional negative time increments. On the other hand, one attractive feature of this process is that its terminal value (${\tilde{C}}_{n}$) is the conventional measure of mean calibration: the difference between the conventional estimate of ATE and the sample average of predicted ITEs.

## 3 Simulation Studies

In this section, we report on proof‐of‐concept simulations that examine the behavior of the proposed approaches. Three sets of simulations were performed. In the first set we assessed the null distribution of *p* values, and in the second and third sets we investigated the power of these tests in detecting various forms of miscalibration. Each simulation scenario was run 10 000 times, a value that results in Monte Carlo standard errors of at most 0.005 for the probability of rejecting $H_{0}$ at 5% significance level (regardless of the actual rejection rate); and SE < 0.003 for the average *p*‐value, thus making the first two digits of rejection probabilities and average *p* values significant. We tested both the one‐part BM test and the two‐part bridge test for both conditional and marginal approaches (four tests in total). We did not compare these tests against any other test, as we are not aware of any other inferential method for our null hypothesis.

All simulation scenarios involve a reference ITE model of the form ($\pi_{a}$ indicates predicted risks under treatment a):

$$\text{logit}\left(\pi_{a}\left(x\right)\right)=\beta_{0}+\beta_{x}x+\beta_{a}a+\beta_{\mathrm{xa}}\mathrm{xa},$$

with x the single predictor, a the binary treatment variable, and Y the binary outcome. Predicted ITEs from this model are generated as $\delta \left(x\right)=\pi_{0}\left(x\right)-\pi_{1}\left(x\right)$.

For the first set of simulations, we generated n simulated individuals given independent draws $x_{i}∼\text{Normal}\left(0,1\right)$, $a_{i}∼\text{Bernoulli}\left(0.5\right)$, $\pi_{i}$ from the reference prediction model, and $Y_{i}∼\text{Bernoulli}\left(\pi_{i}\right)$. In a factorial design, we changed the parameters of the reference model as follows: n∈{500,5000}, $\beta_{0}\in \left\{-1,1\right\}$, $\beta_{x}\in \left\{0,0.25\right\}$, $\beta_{a}\in \left\{-0.5,-0.25\right\}$, and $\beta_{\mathrm{xa}}\in \left\{0,0.25\right\}$.

Table 1 reports, for each of the four tests across all 32 null scenarios, the mean *p*‐value and the empirical rejection rate at the 0.05 significance level. As the distribution of *p* values should be uniform under $H_{0}$, the mean *p*‐value should be close to 0.5 and the rejection rate close to its nominal level. This is generally the case for all four tests, even in scenarios with relatively small samples for ITE model validation. The corresponding empirical cumulative distribution functions of the *p* values, which characterize behavior across the full range of significance levels, are provided in Section S1.

**TABLE 1:** Mean *p*‐value and empirical rejection rate at the 0.05 significance level under $H_{0}$ across the null scenarios, for the four tests^a^.

Scenario					Mean *p*‐value				Rejection rate at α=0.05			
n	$\beta_{0}$	$\beta_{x}$	$\beta_{a}$	$\beta_{\mathrm{xa}}$	BM‐conditional	Bridge‐conditional	BM‐marginal	Bridge‐marginal	BM‐conditional	Bridge‐conditional	BM‐marginal	Bridge‐marginal
500	−1.00	0.00	−0.50	0.00	0.512	0.516	0.509	0.511	0.047	0.048	0.049	0.050
500	−1.00	0.00	−0.50	0.25	0.520	0.522	0.517	0.517	0.045	0.043	0.047	0.046
500	−1.00	0.00	−0.25	0.00	0.512	0.515	0.510	0.511	0.047	0.045	0.048	0.046
500	−1.00	0.00	−0.25	0.25	0.509	0.512	0.506	0.507	0.047	0.045	0.049	0.048
500	−1.00	0.25	−0.50	0.00	0.514	0.517	0.512	0.513	0.048	0.047	0.049	0.049
500	−1.00	0.25	−0.50	0.25	0.514	0.514	0.508	0.506	0.047	0.047	0.049	0.051
500	−1.00	0.25	−0.25	0.00	0.506	0.510	0.505	0.505	0.047	0.045	0.048	0.047
500	−1.00	0.25	−0.25	0.25	0.507	0.511	0.502	0.503	0.046	0.046	0.050	0.047
500	1.00	0.00	−0.50	0.00	0.515	0.515	0.512	0.510	0.046	0.045	0.046	0.048
500	1.00	0.00	−0.50	0.25	0.516	0.519	0.515	0.515	0.047	0.044	0.048	0.047
500	1.00	0.00	−0.25	0.00	0.514	0.518	0.512	0.513	0.051	0.044	0.052	0.049
500	1.00	0.00	−0.25	0.25	0.512	0.516	0.512	0.513	0.047	0.047	0.047	0.048
500	1.00	0.25	−0.50	0.00	0.511	0.515	0.509	0.511	0.050	0.046	0.049	0.048
500	1.00	0.25	−0.50	0.25	0.511	0.512	0.512	0.511	0.051	0.049	0.052	0.050
500	1.00	0.25	−0.25	0.00	0.516	0.518	0.515	0.514	0.044	0.046	0.045	0.050
500	1.00	0.25	−0.25	0.25	0.517	0.521	0.517	0.518	0.047	0.044	0.047	0.046
5000	−1.00	0.00	−0.50	0.00	0.507	0.510	0.506	0.509	0.047	0.048	0.047	0.049
5000	−1.00	0.00	−0.50	0.25	0.504	0.504	0.505	0.505	0.050	0.049	0.049	0.048
5000	−1.00	0.00	−0.25	0.00	0.501	0.503	0.501	0.502	0.050	0.051	0.051	0.050
5000	−1.00	0.00	−0.25	0.25	0.505	0.501	0.505	0.502	0.046	0.046	0.046	0.046
5000	−1.00	0.25	−0.50	0.00	0.508	0.507	0.508	0.507	0.046	0.045	0.048	0.046
5000	−1.00	0.25	−0.50	0.25	0.508	0.508	0.506	0.508	0.049	0.051	0.047	0.050
5000	−1.00	0.25	−0.25	0.00	0.507	0.507	0.507	0.507	0.048	0.047	0.049	0.048
5000	−1.00	0.25	−0.25	0.25	0.504	0.504	0.505	0.505	0.050	0.047	0.048	0.048
5000	1.00	0.00	−0.50	0.00	0.503	0.505	0.502	0.504	0.052	0.052	0.051	0.051
5000	1.00	0.00	−0.50	0.25	0.505	0.507	0.506	0.508	0.046	0.045	0.046	0.044
5000	1.00	0.00	−0.25	0.00	0.505	0.503	0.505	0.503	0.046	0.050	0.048	0.050
5000	1.00	0.00	−0.25	0.25	0.502	0.504	0.503	0.505	0.050	0.046	0.049	0.046
5000	1.00	0.25	−0.50	0.00	0.506	0.506	0.507	0.506	0.045	0.043	0.045	0.044
5000	1.00	0.25	−0.50	0.25	0.505	0.506	0.508	0.509	0.051	0.049	0.050	0.050
5000	1.00	0.25	−0.25	0.00	0.500	0.503	0.500	0.503	0.051	0.049	0.052	0.050
5000	1.00	0.25	−0.25	0.25	0.505	0.503	0.507	0.506	0.049	0.048	0.050	0.047

*Note:* Under $H_{0}$ the *p* values are uniformly distributed, so the mean *p*‐value is expected to be near 0.5 and the rejection rate near 0.05. Each cell is based on 10 000 simulation replications.

^a^ The Monte Carlo standard error is at most 0.003 for the mean *p*‐value and 0.002 for the rejection rate. Scenario columns give the sample size n and the coefficients of the reference model.

While our null hypothesis demands the moderate calibration of predicted ITEs, the conditional approach, as discussed earlier, relies on the condition that predicted baseline risks are calibrated within the strata of predicted ITEs. The condition is satisfied in all above simulations. To explore how the conditional test is affected when this condition is violated, we extend the above simulations, as described in Section S1. Results indicate that both the BM and bridge conditional tests become anti‐conservative (with inflated type I error rate) regardless of the direction of miscalibration. This is an expected finding given that the moments of the martingale (Equations 7 and 9) depend on baseline risks, and thus miscalibrated predicted risks can cause the path of the constructed random walk to deviate from its expectation.

The second and third sets of simulations considered the power of these tests under logit‐linear (second set) and power (third set) transformation of risks to create miscalibrated models. These two sets consider various forms of miscalibration, including those where predicted ITEs are systematically under‐ and over‐estimates, as well as where predicted ITEs are underestimates in some regions and overestimates in others. These scenarios span both directions of model misspecification: cases where the predicted model is simpler than the data‐generating truth, and cases where the predicted model is more flexible than the truth (e.g., scenario s12, in which the model predicts treatment‐effect heterogeneity that is absent from the data‐generating process). The general design features modified versions of our previous simulation setup for nonparametric tests of predicted risks [13]. Here, we consider the reference prediction model as having the following set of parameters: $\beta_{0}=0,\beta_{x}=0.25,\beta_{a}=-0.5,\beta_{\mathrm{xa}}=0.25$. We generate predictor values, treatment assignments, and predicted risks as in the previous setup. Each scenario was run with n∈{500,2500,10000}.

For the second set of simulations, we generate true outcome risks for individuals under treatment as follows:

$$\text{logit}\left(P\left(Y=1|x,a\right)\right)=\beta_{0}+\beta_{x}x+\alpha a+\gamma \left[\beta_{a}a+\beta_{\mathrm{xa}}\mathrm{xa}\right].$$

**FIGURE 3:**
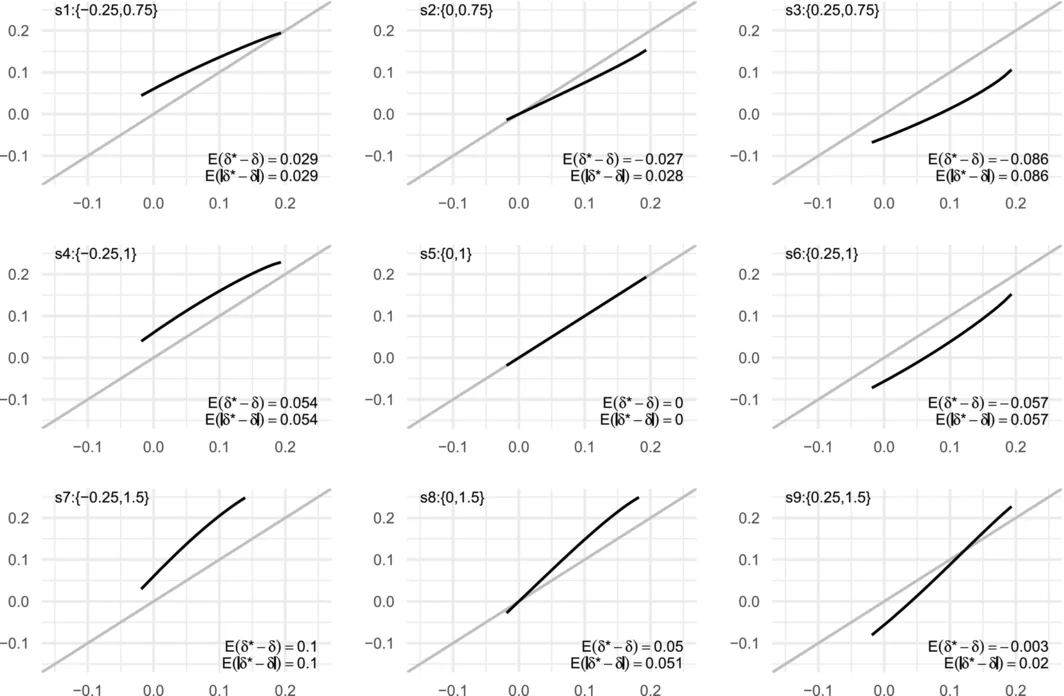
True calibration plots (actual risks on the Y axis and predicted risks on the X axis) for the second set of simulations.

**FIGURE 4:**
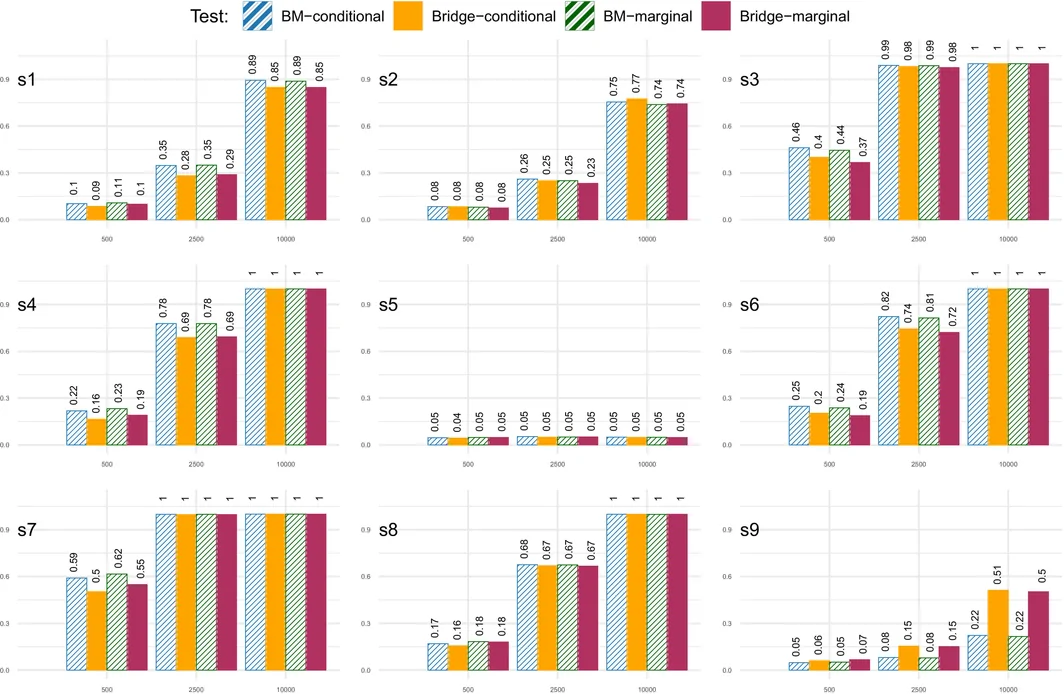
Proportion of significant *p* values (< 0.05) for the second set of simulations. Each bar is based on 10 000 replications; the Monte Carlo standard error of each estimated rejection proportion is at most 0.005 (largest at a proportion of 0.5).

Note that the miscalibrated risks are linear on the logit scale, with the two parameters α and γ controlling, respectively, the location and scale shifts of true risks among the treated. The predicted and true risks among the control group remain equal. The following parameter combinations for {α,γ} were examined: {−0.25, 0.75}, {0, 0.75}, {0.25, 0.75}, {−0.25, 1}, {0, 1}, {0.25, 1}, {−0.25, 1.5}, {0, 1.5}, {0.25, 1.5}. The true calibration plots are provided in Figure 3. Two summary measures are provided on each panel that quantify the extent of miscalibration: the mean calibration error ($E\left(\delta^{*}-\delta \right)$) and the mean absolute calibration error ($E|\delta^{*}-\delta |$). The rejection probabilities (at 5% significance level) as a function of sample sizes are provided in Figure 4.

**FIGURE 5:**
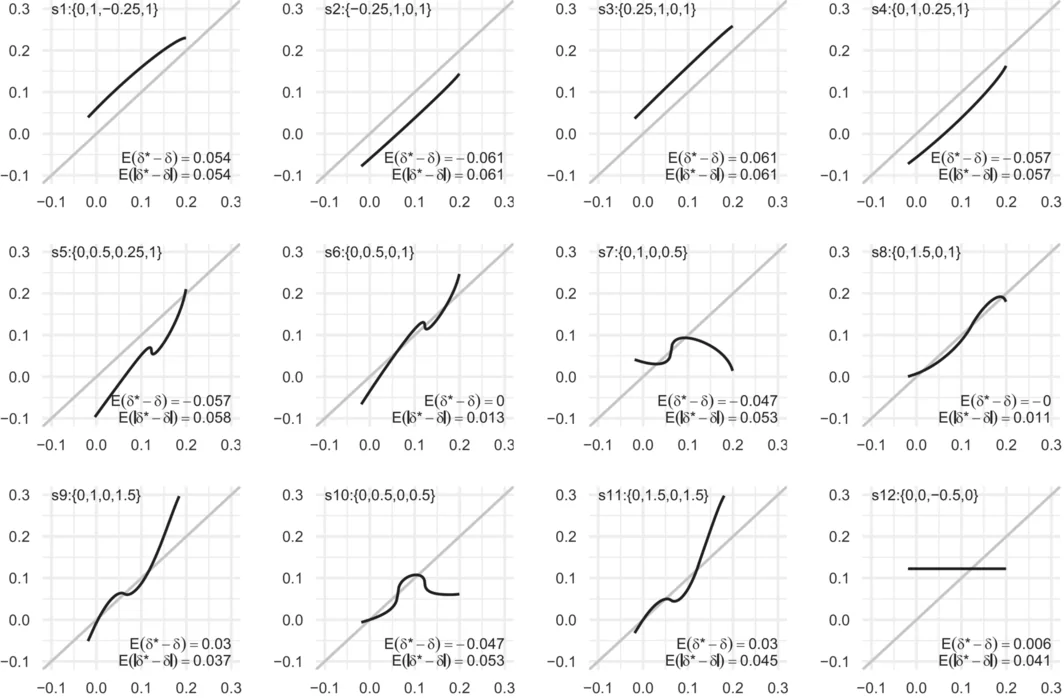
True calibration plots (actual risks on the Y axis and predicted risks on the X axis) for the third set of simulations.

**FIGURE 6:**
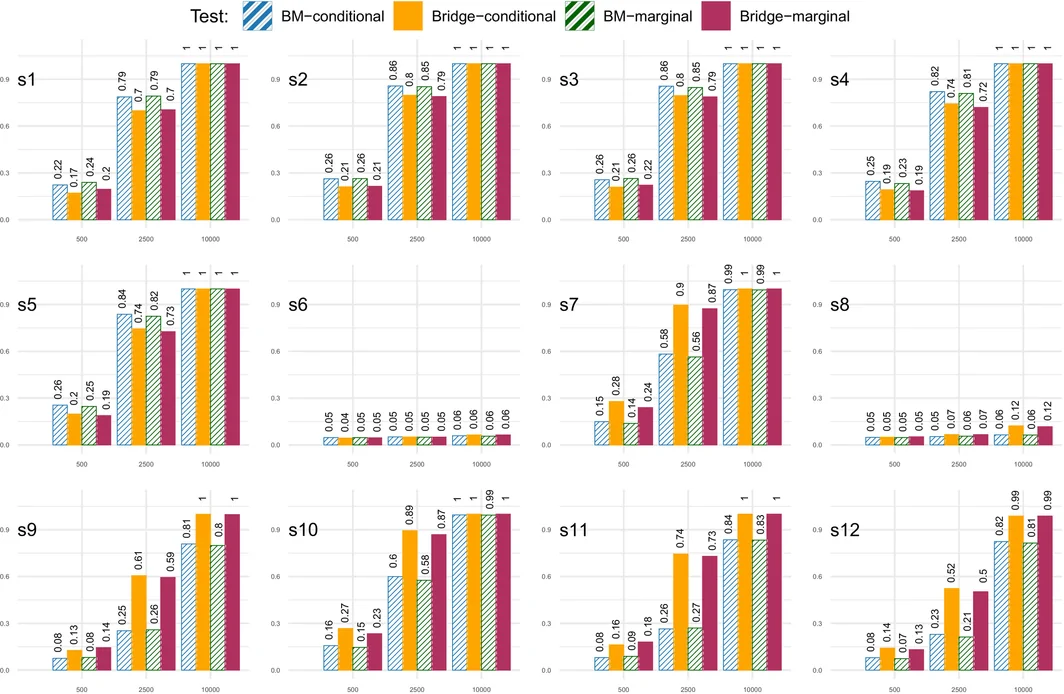
Proportion of significant *p* values (< 0.05) for the third set of simulations. Each bar is based on 10 000 replications; the Monte Carlo standard error of each estimated rejection proportion is at most 0.005 (largest at a proportion of 0.5).

The third set of simulations considered nonlinear forms of miscalibration:

$$\text{logit}\left(P\left(Y=1|x,a\right)\right)=\alpha_{a}+\gamma_{a}\mathrm{sign}\left(\text{logit}\left(\pi_{a}\left(x\right)\right)\right){\left|\text{logit}\left(\pi_{a}\left(x\right)\right)\right|}^{\gamma_{a}}$$

here $\alpha_{a}$ introduces bias to the predicted risk while the terms involving $\gamma_{a}$ create an odd function that introduces nonlinear miscalibration. This function was applied to predicted risks within each treatment arm, resulting in four degrees of freedom. The following parameter combinations for $\left\{\alpha_{0},\gamma_{0},\alpha_{1},\gamma_{1}\right\}$ were examined: {0, 1, −0.25, 1}, {−0.25, 1, 0, 1}, {0.25, 1, 0, 1}, {0, 1, 0.25, 1}, {0, 0.5, 0.25, 1}, {0, 0.5, 0, 1}, {0, 1, 0, 0.5}, {0, 1.5, 0, 1}, {0, 1, 0, 1.5}, {0, 0.5, 0, 0.5}, {0, 1.5, 0, 1.5}, {0, 0, −0.5, 0}. In the last scenario (s12), the ITE model falsely predicts treatment effect heterogeneity. The true calibration plots for these scenarios are provided in Figure 5. Results of these simulations are provided in Figure 6.

Overall, across two sets of simulations, involving different forms and degrees of miscalibrations, the tests demonstrated the expected behavior: detecting miscalibrations with a power that generally increases with the severity of miscalibration and the sample size. Some other patterns are worth highlighting. The tests had low power for detecting miscalibrated ITEs where the calibration function was close to the identity line, even though the underlying predicted risks within each arm were highly miscalibrated (e.g., scenarios s6 and s8 in the second set). The conditional and marginal tests had nearly identical power. This was not only the case in the second set (where baseline predicted risks remained calibrated), but also in the third set, where in many scenarios the baseline risks were miscalibrated. The two‐part bridge tests had generally non‐inferior power compared with their one‐part BM counterparts, and had more power in scenarios where the miscalibration function was highly non‐linear (e.g., scenarios s7 and s9–s12 in the third set). The scenarios where the one‐part tests had marginally more power (s4, s6 in the second set, s1–s5 in the third set) all pertain to cases where the calibration function is (almost) linear. The bridge component of the two‐part test is sensitive to nonlinear forms of miscalibration. As such, in these scenarios, this component is generally non‐significant, resulting in the *p*‐value of Fisher's method moving away from significance.

## 4 Case Study

We use data from the GUSTO‐I study, which is a well‐explored dataset in predictive analytics methodological research [31]. GUSTO‐I was a multi‐national clinical trial of streptokinase (SK) and tissue plasminogen activator (TPA), two antithrombotic medications used for immediate management of heart attacks. The primary endpoint was mortality risk within 30 days. Given that death status could be verified for all, there were no censored observations. The original study had four study arms (SK + subcutaneous heparin, SK + intravenous heparin, TPA + intravenous heparin, and SK + TPA + intravenous heparin). Combined SK + TPA is not currently recommended due to high risk of bleeding, and the original study ultimately combined the two SK groups (which showed similar effects). As such, for this case study, we consider tPA alone versus SK therapy, regardless of the mode of delivery of concomitant heparin. We use this dataset to estimate ITEs for TPA versus SK therapy after a heart attack. We assess the out‐of‐sample performance of the resulting ITEs using the proposed visualizations, summary metrics, and inference. All results are generated using the *cumulcalib* R package that accompanies this paper (https://github.com/resplab/cumulcalib), with exemplary code provided in Section S2.

Similar to previous case studies, we use the non‐US subset of the sample to develop the ITE models, and use the US sub‐sample to evaluate it. As this case study is an illustration, we will develop two ITE models. The large‐development‐sample model uses the entire development sample (n= 13 342), while the small‐development‐sample model is based on a random subset consisting of 10% of the development sample. Similarly, we use two validation samples. The large validation sample uses the entire validation sample (n= 17 168), while the small validation sample uses a random subset consisting of 10% of the validation sample. Outcome risks (the probability of dying in the first 30 days after the event) are as follows: 0.074 for the large development sample, 0.069 for the small development sample, 0.067 for the large validation sample, and 0.061 for the small validation sample. The corresponding ATEs were 0.007, −0.017, 0.012, and 0.024.

We fit models of the form $\text{logit}P\left(Y=1\right)=\beta_{0}+\beta_{1}\left[\text{female}\right]+\beta_{2}\left[\mathrm{age}\right]+\beta_{3}\left[\mathrm{AMI}\;\text{location}-\text{other}\right]+\beta_{4}\left[\mathrm{AMI}\;\text{location anterior}\right]+\beta_{5}\left[\text{previous}\;\mathrm{AMI}\right]+\beta_{6}\left[\text{Killip score}\right]+\beta_{7}\left[\min\left(\text{systolic blood pres}\text{sure},100\right)\right]+\beta_{8}\left[\text{pulse rate}\right]+\beta_{9}\left[a\right]+\beta_{10}\left[a:\text{female}\right]+\beta_{11}\left[a:\mathrm{age}\right]$, with a indicating treatment and a:b indicating the first‐order interaction between two predictors. Note that the treatment indicator (TPA: 1, SK: 0) enters the model both as a main effect and with first‐order interaction with sex and age (reflecting our hypothesis that these variables are treatment effect modifiers on the logit scale). The coefficients of these models are provided in the footnotes of the cumulative calibration plots below.

### 4.1 Large‐Development‐Sample Model

**FIGURE 7:**
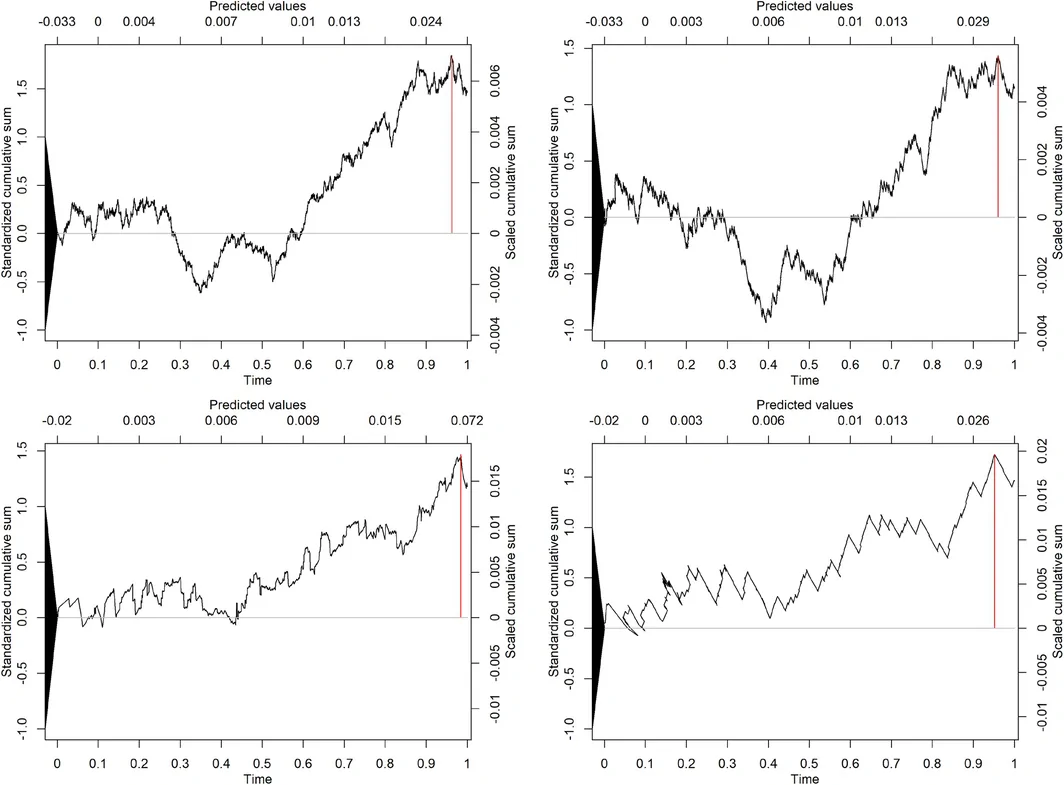
Visualization of the {S} process for the large‐development‐sample model, an example whose cumulative calibration plot shows no systematic departure from calibration. Top row: Large validation sample; bottom row: Small validation sample. Left column: Conditional approach; right column: Marginal approach. *How to read these plots:* The primary (bottom and left) axes show the time and location components of the standardized {S} process (Equation 3). Under moderate calibration this path behaves like a standard Brownian motion, fluctuating randomly around zero with no systematic drift, whereas a systematic drift or a large excursion indicates miscalibration. The secondary (top) X‐axis shows the predicted ITE (δ), which maps one‐to‐one to time; the secondary (right) Y‐axis shows the scaled, un‐standardized process, whose terminal value is $C_{n}$ (the mean calibration error). The solid vertical line marks the location and direction of the maximum absolute cumulative calibration error, $C^{*}$. Model coefficients ($\beta_{0}$–$\beta_{11}$) are, respectively: −1.2246, 0.3147, 0.0684, 0.3926, 0.5353, 0.4596, 0.7375, −0.0790, 0.0179, −0.8846, −0.0405, 0.0111.

Figure 7 presents the visualization of the {S} process for this model for the large (top row) and small (bottom row) validation samples, for the conditional (left column) and marginal (right column) approaches. Details of this visualization are similar to those proposed for the risk prediction context [13]. The primary X‐ and Y‐axes pertain to, respectively, time and location components of the standardized ({S}) process. The secondary (top) X‐axis is utilized to show the predicted ITEs (δs), noting that there is a 1:1 mapping between a given time value and a predicted value. The secondary (right) Y‐axis shows the scaled (but not standardized) process; the terminal value of the random walk on this axis corresponds to $C_{n}$, providing a convenient visual assessment of mean calibration error. The solid vertical line marks the location and direction of the maximum absolute cumulative calibration error ($C^{*}$), the point at which the standardized process is farthest from zero. Note the occasional negative jumps in the marginal graph for the small validation sample (bottom‐right panel).

The values of $C_{n}$ and $C^{*}$, and the *p*‐value for the null hypothesis that the model is moderately calibrated, are reported in Table 2. For example, in the large validation sample under the conditional approach, $C_{n}=0.0055$ and $C^{*}=0.0070$ (on the risk‐reduction scale). Overall, the figures and summary indices are compatible with this ITE model being calibrated: the random walk does not drift systematically from its expected path. *p* values indicate that the data are not strongly against the claim that the model is calibrated in either sample.

**TABLE 2:** Large‐development‐sample model validation results.

Validation sample	Approach	$C_{n}$ (mean calibration error)	$C^{*}$ (maximum absolute cumulative error)	*p*‐value(bridge test)
Large	Conditional	0.0055	0.0070	0.0633
Large	Marginal	0.0045	0.0056	0.0554
Small	Conditional	0.0144	0.0179	0.5432
Small	Marginal	0.0166	0.0196	0.4054

*Note:* $C_{n}$ is the (signed) mean calibration error and $C^{*}$ the (non‐negative) maximum absolute cumulative calibration error, both on the risk‐reduction scale. The *p*‐value is that of the two‐part bridge test (the combination, via Fisher's method, of its mean‐calibration and bridge components).

### 4.2 Small‐Development‐Sample Model

**FIGURE 8:**
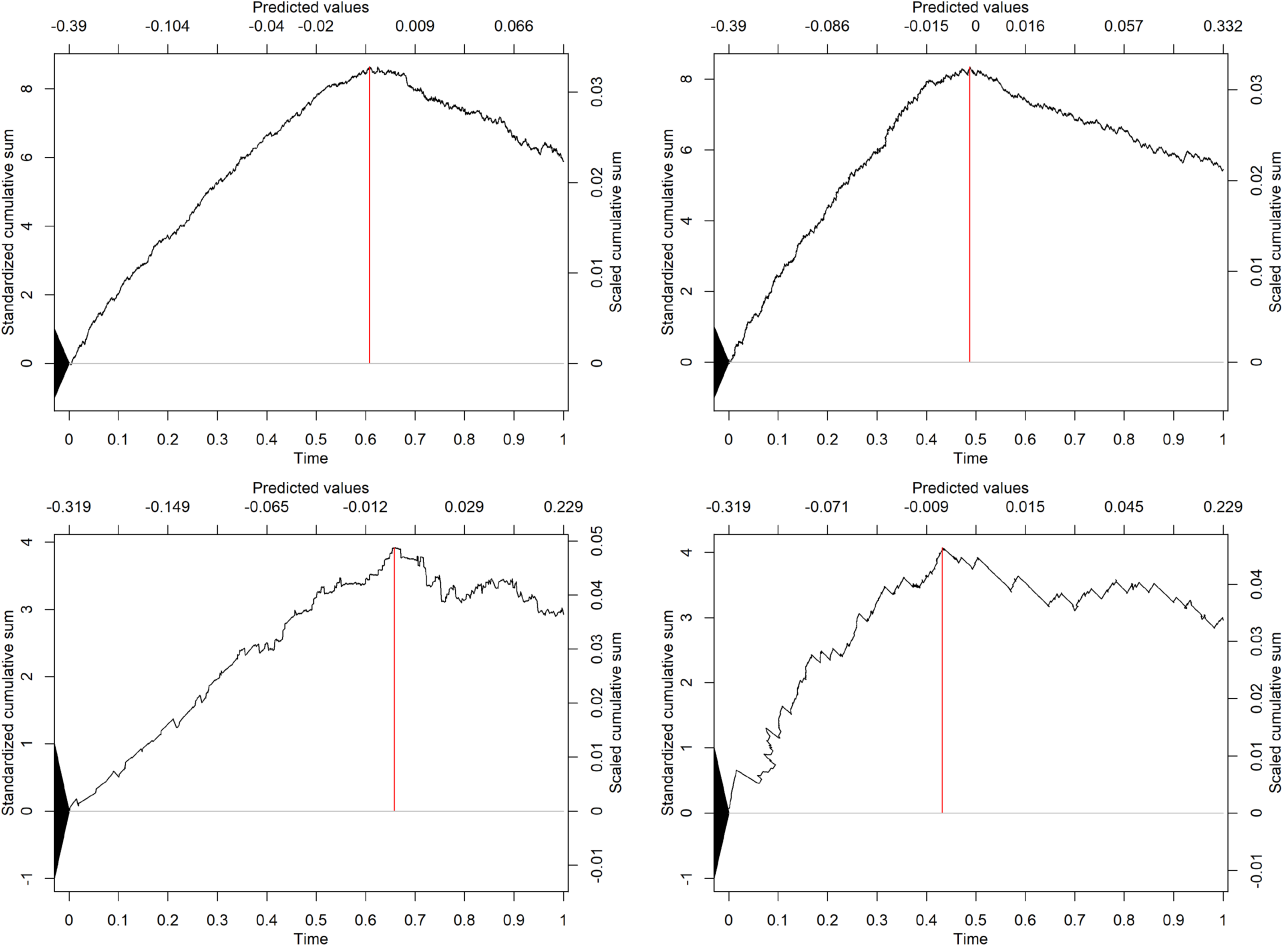
Visualization of the {S} process for the small‐development‐sample model, an example of a clearly miscalibrated ITE model. Rows and columns are as in Figure 7. Axes and plot elements are as described in Figure 7. Here the standardized path rises and then falls (an inverted U): Prediction errors accumulate positively for small predicted ITEs (benefit under‐estimated) and negatively for large ones (benefit over‐estimated)—the signature of an over‐fitted model that exaggerates the spread of predicted ITEs. The large $C^{*}$ and small bridge‐test *p* values (Table 3) confirm strong evidence of miscalibration. Model coefficients ($\beta_{0}$–$\beta_{11}$) are, respectively: −2.2635, 0.4721, 0.0529, −0.5550, 0.4923, 0.4515, 0.6840, −0.0622, 0.0210, −2.3426, −1.4945, 0.0435.

Graphical and numerical results are provided in Figure 8 and Table 3, respectively. Here, the model is clearly miscalibrated in both validation samples. The prediction errors accumulate on the left side of the graph (up to around a predicted ITE of 0, as indicated by the secondary X‐axis), indicating that the model under‐estimates the actual benefit when the predicted ITE is small (actual ITE > predicted ITE, resulting in positive errors). The curve then declines, indicating that the model over‐estimates the benefit for larger predicted ITEs (actual ITE < predicted ITE, resulting in negative errors). As noted earlier, this is a signature of an over‐fitted model that makes exaggerated predictions: it over‐predicts benefit for patients with high predicted ITE and under‐predicts it for those with low predicted ITE. Correspondingly, the summary indices are large, indicating a substantial degree of miscalibration, and the bridge *p* values are small, indicating strong evidence for miscalibration. In such instances, the location of $C^{*}$ provides useful auxiliary information: taking the conditional approach in the large validation sample as an example, the maximum absolute cumulative deviation occurs at a predicted ITE of −0.0034. Predicted ITEs around this value are approximately calibrated, whereas those below it are downwardly biased (benefit under‐estimated) and those above it are upwardly biased (benefit over‐estimated).

**TABLE 3:** Small‐development‐sample model validation results.

Validation sample	Approach	$C_{n}$ (mean calibration error)	$C^{*}$(maximum absolute cumulative error)	*p*‐value (bridge test)
Large	Conditional	0.0223	0.0328	< 0.0001
Large	Marginal	0.0212	0.0325	< 0.0001
Small	Conditional	0.0364	0.0488	< 0.0001
Small	Marginal	0.0337	0.0464	< 0.0001

*Note:* Columns are as in Table 2: $C_{n}$ is the (signed) mean calibration error and $C^{*}$ the (non‐negative) maximum absolute cumulative calibration error (both on the risk‐reduction scale), and the *p*‐value is that of the two‐part bridge test.

## 5 Discussion

In this work we proposed novel nonparametric methods for the assessment of moderate calibration of predicted ITEs for binary outcomes. These methods are applicable when a pre‐specified ITE model is being validated in a new sample. We have demonstrated that moderate calibration can be assessed without resorting to regularization if calibration is examined on the cumulative sum domain. We proposed an estimator for cumulative ITE errors and showed that a properly scaled version of this estimator will result in a stochastic process that, under the null hypothesis that the ITE model is moderately calibrated, converges point‐wise to the standard Brownian motion. We showed that the conditional moments of this estimator depend on the true outcome risks. For ITE models that predict risk, such predictions can be used under the condition that they are calibrated, giving rise to our proposed “conditional” approach. However, the investigator might not be willing to assume those risks are calibrated; indeed, not all ITE models generate predicted risks. For these instances, we proposed an approximate “marginal” approach that solely relies on predicted ITEs. These developments enabled us to extend the previously proposed visualization, summarizing indices, and tests for moderate calibration of predicted risk to the predicted ITE domain. In a case study we laid out our suggestions for the visualization of cumulative prediction errors and the interpretation of proposed tests. The proposed tests are implemented in the *cumulcalib* R package (https://cran.r‐project.org/package=cumulcalib) with exemplary code provided in Section S2.

For inference, our proof‐of‐concept simulations confirmed the desired properties of the resulting tests. Simulations under the null hypothesis, where ITE predictions were moderately calibrated, showed that these asymptotic tests generally maintain their type I error rate, even with sample sizes of 500, which we consider modest for an ITE model validation study. In simulations where the null hypothesis was violated, the power of these tests increased with larger sample sizes and with more severe miscalibrations, albeit the power could be low if the true calibration function was close to the identity line. The two‐part (bridge) test had generally more power than the one‐part test (at times significantly), an unsurprising finding given that this test offers two independent opportunities for detecting miscalibration. Because it rests on between‐arm differences, assessing ITE calibration is more demanding, in terms of sample size, than assessing risk calibration. This is related to the notion that detecting treatment‐by‐covariate interaction requires significantly larger sample sizes [32]. In our simulations the tests were well‐powered at n=10,000 and detected pronounced miscalibration at n=2,500, but had limited power at n=500. In modest samples, therefore, high *p* values, especially in the presence of a large $C^{*}$, should not be interpreted as evidence in favor of calibration; rather, that the sample does not offer strong evidence against the claim that the model is calibrated. We generally advise against significance testing at an arbitrary level for model validation. Rather, *p* values are better interpreted as continuous measures of the compatibility between the data and the hypothesis of moderate calibration, complementing, not replacing, the reported magnitude of miscalibration [33].

How can these developments be used in practice? Some models generate ITEs from underlying predicted risks under alternative treatment decisions. Such predicted risks might be used for decision making in tandem with predicted ITEs. Correspondingly, one might demand that the model generate calibrated predictions across all its outputs. Requiring an ITE model to be calibrated on both predicted risk and ITE domains is equal to requiring it to be calibrated on predicted risks within each treatment group. One option is therefore to assess calibration separately for risks within each treatment group. However, this does not put the focus on calibration of ITEs, which might be of independent interest. Indeed, miscalibration of risks within each group might cancel out and result in calibrated ITEs; reciprocally, otherwise subtle miscalibrations of risks can augment and result in more pronounced miscalibration of ITEs. These patterns might not be obvious when predicted risks are assessed independently. Therefore, even for ITE models that also predict risk we propose an assessment of the calibration of ITEs, especially the plot of the {S} process. If the predicted risks are deemed calibrated (or the model is modified to correct for miscalibration), one can then use the conditional approach, for which a functional CLT could be proven. However, the marginal approach also seems to have desirable properties while not requiring the assumption of calibrated predicted risks. The latter remains the only option for models that generate predicted ITEs without modeling risks.

We propose visualization of the standardized cumulative calibration plot, as well as reporting $C_{n}$, that is, mean calibration error, and $C^{*}$, that is, maximum absolute cumulative prediction error. If inference is desired, we suggest the use of the two‐part “bridge” test over the one‐part BM test (all these suggestions are exemplified in our case study). Investigators often assess mean calibration (by comparing the predicted and observed ATEs) before moderate calibration. As mean calibration is a necessary condition for moderate calibration, such sequential inference alters the power of assessing moderate calibration. The bridge test provides an opportunity for joint inference that preserves the desired overall type I error rate while reducing type II error rate because of its higher power. Further, in many instances, if mean calibration assessment is not satisfactory, one might first decide to address the miscalibrated ATEs before embarking on moderate calibration assessment. For example, for an ITE model that predicts risks under control and intervention strategies using a logit link function, one might fix a biased ATE prediction by adjusting the intercept of the logit functions separately within each treatment arm. This approach results in average predicted risks exactly matching the average observed risks within treatment groups, in turn leading to an exact match between the predicted and observed ATEs. In these instances, the one‐part BM test is not a valid inference method, as the terminal value of the {S} process is guaranteed to be zero. In such instances, one can apply the bridge component of the bridge test in isolation to assess moderate calibration conditional on mean calibration having been achieved.

There are several areas for future research. As a first step, we proposed these approaches for binary outcomes and when the sample is from a randomized experiment. An important extension, with wide applicability, would be for other outcome types, in particular survival outcomes. For such outcomes, the challenge is that the observed response is not only a function of predicted risk, but also the distribution of censoring events and the shape of the hazard function. As for extending this approach to non‐randomized samples, the challenge is that a, δ, and Y can be dependent in such settings, violating the martingale properties underpinning the proposed stochastic processes. A potential path forward is the application of inverse‐probability‐of‐treatment weighting schemes that can result in pseudo‐populations where treatment assignment can be considered random. However, the application of a functional CLT when observations are weighted would require additional technical developments. Further, other known properties of the Brownian motion can be used to propose alternative tests. In addition to the test based on maximum absolute deviation, Arrieta‐Ibarra et al. [21] proposed a test based on the maximum range of the Brownian motion (max(W)−min(W)). In the same vein, a test based on the maximum range of the Brownian bridge will result in a new two‐part test. Another potential test would be based on the square of the area under the Brownian motion and Brownian bridge. The resulting tests might have more power as they capture the behavior of the {S} process across its entire path. Lastly, our simulation studies were aimed at investigating the expected properties of the proposed tests. The power of these tests in detecting specific types of miscalibrations should be investigated in dedicated simulation studies. This should include a deeper investigation of the consequences of predicted risks not being calibrated for the conditional approach, and investigating the marginal approach in more varied settings given its heuristic underpinnings. Relatedly, while our framework conditions on the model's predicted ITEs and is thus agnostic to how they were generated (and the proposed marginal approach does not require arm‐specific predicted risks to be available), our simulations produced predicted ITEs as differences of arm‐specific predicted risks. Assessing predictions from estimators that target the treatment effect directly (e.g., causal forests or R‐learners) is a further useful direction for future work.

A further extension of this framework is to study calibration of predictions across the domain of another variable. Conventionally, as in this work, we assess calibration as the closeness of predicted and actual quantities across the strata of predicted quantities (ITEs in our case). However, this closeness can also be investigated across the strata of another variable. For example, it is often reasonable to investigate whether a risk prediction or an ITE model makes calibrated predictions among individuals of different age. This involves assessing the closeness of predicted and observed risks or ITEs within strata defined by age. The core idea of nonparametric calibration assessment is to transport the conditional mean $E\left(Y^{\left(0\right)}-Y^{\left(1\right)}-\delta |\delta =z\right)$ into the cumulative domain $E\left(Y^{\left(0\right)}-Y^{\left(1\right)}-\delta |\delta <z\right)$. The premise is that the latter quantity can be estimated, with proper scaling, from the cumulative sums of prediction errors, once ordering the sample from the small to large values of δ. Imagine we are now interested in assessing the calibration of predictions across the strata of a continuous variable h (e.g., age). The quantity of interest therefore becomes $E\left(Y^{\left(0\right)}-Y^{\left(1\right)}-\delta |h=z\right)$, which again can be cast onto the cumulative sum domain $E\left(Y^{\left(0\right)}-Y^{\left(1\right)}-\delta |h<z\right)$. The latter can be estimated via scaled cumulative sums, this time after ordering the observations (δ,a,Y) from the small to large values of h. The subsequent steps will remain as described in this work, including visualization and inference. This form of calibration assessment is especially relevant for ITE models that combine predicted risks with an estimate of treatment effect. As an example, the ACCEPT model, a risk prediction model for exacerbations in patients with obstructive lung disease [34], is also used to predict the ITE (risk reduction) of treatment with azithromycin. This is achieved by applying a constant treatment effect, estimated from relevant RCTs, to predicted risks. It might be of interest to check if the assumption of a constant effect holds across the strata of predicted risks. For this purpose, once the observations in the sample are ordered from small to large values of predicted risks, the {S} process can be constructed as before.

Presently, most prediction models focus on outcome risk under a single control treatment (e.g., standard of care). Even though these models are not designed to individualize treatment decisions, they are often used as such, typically by assuming that individuals at high outcome risk should be prioritized for active treatment. On the other hand, by predicting individualized values of expected treatment effect, ITE models provide a rational framework for medical decision‐making. Perhaps due to recognizing the importance of ITE models, and the increasing availability of data to support their development, there is a rising interest in both the methodology and application of such models. Calibration is an often overlooked aspect of prediction models in general [35], but is arguably even more critical for predicted ITEs due to their direct relevance in informing treatment decisions. Therefore, it seems timely to highlight the importance of calibration for ITE models and promote objective, reproducible methods for its assessment.

## Funding

This research was supported by The Canadian Institutes of Health Research—Team Grant PHT 178432.

## Conflicts of Interest

The authors declare no conflicts of interest.

## Supporting information


**Section S1:** Additional simulations.
**Section S2:** Exemplary R code.
**Section S3:** Code and data availability.

## Data Availability

The data that support the findings of this study are openly available in the cumulcalibbenefit folder at https://github.com/resplab/papercode/.

## Appendix 1 Equations for Increments and Their Conditional Expectations for $B_{k}$

$$D_{k}=B_{k}-B_{k-1}=\left\{\begin{array}{lll} k\frac{\sum_{i=1}^{k}\left(1-a_{i}\right)Y_{i}}{\sum_{i=1}^{k}\left(1-a_{i}\right)}-\left(k-1\right)\frac{\sum_{i=1}^{k-1}\left(1-a_{i}\right)Y_{i}}{\sum_{i=1}^{k-1}\left(1-a_{i}\right)}-\frac{\sum_{i=1}^{k-1}a_{i}Y_{i}}{\sum_{i=1}^{k-1}a_{i}} & & a_{k}=0\\ \frac{\sum_{i=1}^{k-1}\left(1-a_{i}\right)Y_{i}}{\sum_{i=1}^{k-1}\left(1-a_{i}\right)}-\left[k\frac{\sum_{i=1}^{k}ta_{i}Y_{i}}{\sum_{i=1}^{k}a_{i}}-\left(k-1\right)\frac{\sum_{i=1}^{k-1}a_{i}Y_{i}}{\sum_{i=1}^{k-1}a_{i}}\right] & & a_{k}=1 \end{array}\right.$$

$$\begin{array}{cc} \;\mu_{k} & =E\left(B_{k}-B_{k-1}|F_{k-1}\right)\\ & \;=\left\{\begin{array}{ll} k\frac{\sum_{i=1}^{k-1}\left(1-a_{i}\right)Y_{i}+\pi_{k}^{*}}{\sum_{i=1}^{k}\left(1-a_{i}\right)}-\left(k-1\right)\frac{\sum_{i=1}^{k-1}\left(1-a_{i}\right)Y_{i}}{\sum_{i=1}^{k-1}\left(1-a_{i}\right)}-\frac{\sum_{i=1}^{k-1}a_{i}Y_{i}}{\sum_{i=1}^{k-1}a_{i}} & a_{k}=0\\ \frac{\sum_{i=1}^{k-1}\left(1-a_{i}\right)Y_{i}}{\sum_{i=1}^{k-1}\left(1-a_{i}\right)}-\left[k\frac{\sum_{i=1}^{k-1}a_{i}Y_{i}+\left(\pi_{k}^{*}-\delta_{k}^{*}\right)}{\sum_{i=1}^{k}a_{i}}-\left(k-1\right)\frac{\sum_{i=1}^{k-1}a_{i}Y_{i}}{\sum_{i=1}^{k-1}a_{i}}\right] & a_{k}=1 \end{array}\right.,\; \end{array}$$

where $\pi_{k}^{*}=E\left(Y|\delta =\delta_{k},a=0\right)$ and $\delta_{k}^{*}=E\left(Y|\delta =\delta_{k},a=0\right)-E\left(Y|\delta =\delta_{k},a=1\right)$.

## Appendix 2 Proof of the Lindeberg Condition

We need to show that

$$\lim_{n\to \infty }\frac{1}{s_{n}^{2}}\sum_{k=1}^{n}\left[E{\left(D_{k}-\mu_{k}\right)}^{2}\;1_{\left\{\;|D_{k}-\mu_{k}|>\epsilon s_{n}\right\}}\right]=0$$

for all ϵ>0, where

$$D_{k}-\mu_{k}=k\left(\left(1-a_{k}\right)\frac{Y_{k}-\pi_{k}^{*}}{\sum_{i=1}^{k}\left(1-a_{i}\right)}-a_{k}\frac{Y_{k}-\pi_{k}^{*}+\delta_{k}^{*}}{\sum_{i=1}^{k}a_{i}}\right),$$

and

$$s_{n}^{2}=\sum_{k=1}^{n}k^{2}\left[\left(1-a_{k}\right)\frac{\pi_{k}^{*}\left(1-\pi_{k}^{*}\right)}{{\left(\sum_{i=1}^{k}\left(1-a_{i}\right)\right)}^{2}}+a_{k}\frac{\left(\pi_{k}^{*}-\delta_{k}^{*}\right)\left(1-\pi_{k}^{*}+\delta_{k}^{*}\right)}{{\left(\sum_{i=1}^{k}a_{i}\right)}^{2}}\right].$$

Our proof is for when individuals are allocated to the treatment or control groups by a random mechanism that is unrelated to the response and the predicted benefits. Let $A_{k}=\sum_{i=1}^{k}a_{i}$. The following regularity conditions are required: $\lim_{n\to \infty }A_{n}/n=\lambda$, where 0<λ<1; and the true conditional risks $\pi_{k}^{*}$ and $\pi_{k}^{*}-\delta_{k}^{*}$ are bounded away from 0 and 1, ensuring $\lim_{n\to \infty }s_{n}=\infty$.

Without loss of generality we assume 0<λ≤0.5. Because the numerators of the fractions in the formula for $D_{k}-\mu_{k}$ are always between −1 and 1, we have

$$|D_{k}-\mu_{k}|<k\left(\left(1-a_{k}\right)\frac{1}{k-A_{k}}+a_{k}\frac{1}{A_{k}}\right)\leq \frac{k}{\min\left(k-A_{k},A_{k}\right)}.$$

Note that the denominator in the nonzero term above counts the subjects, among the first k, in the same arm as the kth subject, and is therefore at least one; hence $|D_{k}-\mu_{k}|<k$. So, defining

$$Z_{k}=\frac{k}{\min\left(k-A_{k},A_{k}\right)},$$

we have

$$E\left[{\left(D_{k}-\mu_{k}\right)}^{2}\;1_{\left\{\;|D_{k}-\mu_{k}|>\epsilon s_{n}\right\}}\right]\leq E\left[{\left(D_{k}-\mu_{k}\right)}^{2}\;1_{\left\{Z_{k}>\epsilon s_{n}\right\}}\right].$$

Therefore, it we establish the limit with the term on the right‐hand side instead, the goal is achieved. Further, as the term $\epsilon s_{n}$ grows without bound, if we change it to any fixed value and prove the result, it will be sufficient. Thus, we show that

$$\lim_{n\to \infty }\frac{1}{s_{n}^{2}}\sum_{k=1}^{n}E\left[{\left(D_{k}-\mu_{k}\right)}^{2}\;1_{\left\{Z_{k}>\lambda^{-1}+\xi \right\}}\right]=0$$

for some fixed ξ>0 (note that $\lim_{k\to \infty }\frac{k}{\min\left(k-A_{k},A_{k}\right)}=\lambda^{-1}$). But

$$\begin{array}{cc} P\left(Z_{k}>\lambda^{-1}+\xi \right) & =P\left(\frac{k}{\min\left(k-A_{k},A_{k}\right)}>\lambda^{-1}+\xi \right)\\ & \;=P\left(\min\left(k-A_{k},A_{k}\right)<\frac{k}{\lambda^{-1}+\xi }\right)\\ & \;\leq P\left(\min\left(2\lambda k-A_{k},A_{k}\right)<\frac{k}{\lambda^{-1}+\xi }\right)\\ & \;=P\left(\min\left(\lambda k-A_{k},A_{k}-\lambda k\right)<\frac{k}{\lambda^{-1}+\xi }-\lambda k\right)\\ & \;=P\left(\;|A_{k}-\lambda k|>k\frac{\lambda^{2}\xi }{1+\lambda \xi }\right). \end{array}$$

Due to the assumed independence between the treatment allocations and the response and predicted benefits, we can assume ordering the sample on predicted benefits (as part of constructing the {S} process) is equivalent to a random permutation of the treatment allocations. As such, by Hoeffding's inequality for sampling without replacement [36] (Section 6),

$$\forall c>0:P\left(\left|A_{k}-kA_{n}/n\right|>\mathrm{kc}\right)\leq 2e^{-2kc^{2}}.$$

Writing $c_{\xi }=\frac{\lambda^{2}\xi }{1+\lambda \xi }$, and noting that $A_{n}/n\to \lambda$ implies $|A_{n}/n-\lambda |\leq c_{\xi }/2$ for all sufficiently large n, we obtain, for all such n and all k≤n,

$$P\left(\;|A_{k}-\lambda k|>kc_{\xi }\right)\leq P\left(\left|A_{k}-kA_{n}/n\right|>kc_{\xi }/2\right)\leq 2e^{-kc_{\xi }^{2}/2}.$$

$$\;\text{Since}\;|D_{k}-\mu_{k}|<k,$$

$$\sum_{k=1}^{n}E\left[{\left(D_{k}-\mu_{k}\right)}^{2}\;1_{\left\{Z_{k}>\lambda^{-1}+\xi \right\}}\right]\leq \sum_{k=1}^{n}k^{2}\;P\left(Z_{k}>\lambda^{-1}+\xi \right)\leq \sum_{k=1}^{\infty }2k^{2}e^{-kc_{\xi }^{2}/2}<\infty ,$$

where the last series converges as the exponential factor dominates the polynomial term (ratio test). As $s_{n}$ grows without bound,

$$\lim_{n\to \infty }\frac{1}{s_{n}^{2}}\sum_{k=1}^{n}E\left[{\left(D_{k}-\mu_{k}\right)}^{2}1_{\left\{Z_{k}>\lambda^{-1}+\xi \right\}}\right]=0.$$
