# Supplementary material for: Nonparametric Assessment of the Calibration of Individualized Treatment Effects
Source: Stat Med. 2026 Aug 25;45(20-22):e70724. doi: 10.1002/sim.70724 (PMC13504708; doi:10.1002/sim.70724)
Supplement: Supplementary file 1 [file SIM-45-0-s001.pdf]

Supplementary Material for 'Non-parametric assessment of the calibration of individualized treatment effects'

August 05, 2026

Table of Contents

Section 1: Additional simulations

Section 2: Exemplary R code (based on cumulcalib package)

Section 3: Code and data availability

Section 1: Additional simulations

Empirical distribution of p-values under the null

Figure 1 presents the empirical cumulative distribution functions (CDFs) of the p-values from the four tests across the null scenarios described in the main text, complementing the rejection rates reported there. Under H_0, p-values are uniformly distributed, so a well-behaved test produces an empirical CDF close to the identity line. This is the case for all four tests, even at the smaller sample size.

Figure 1: Empirical CDF of p-values under H_0

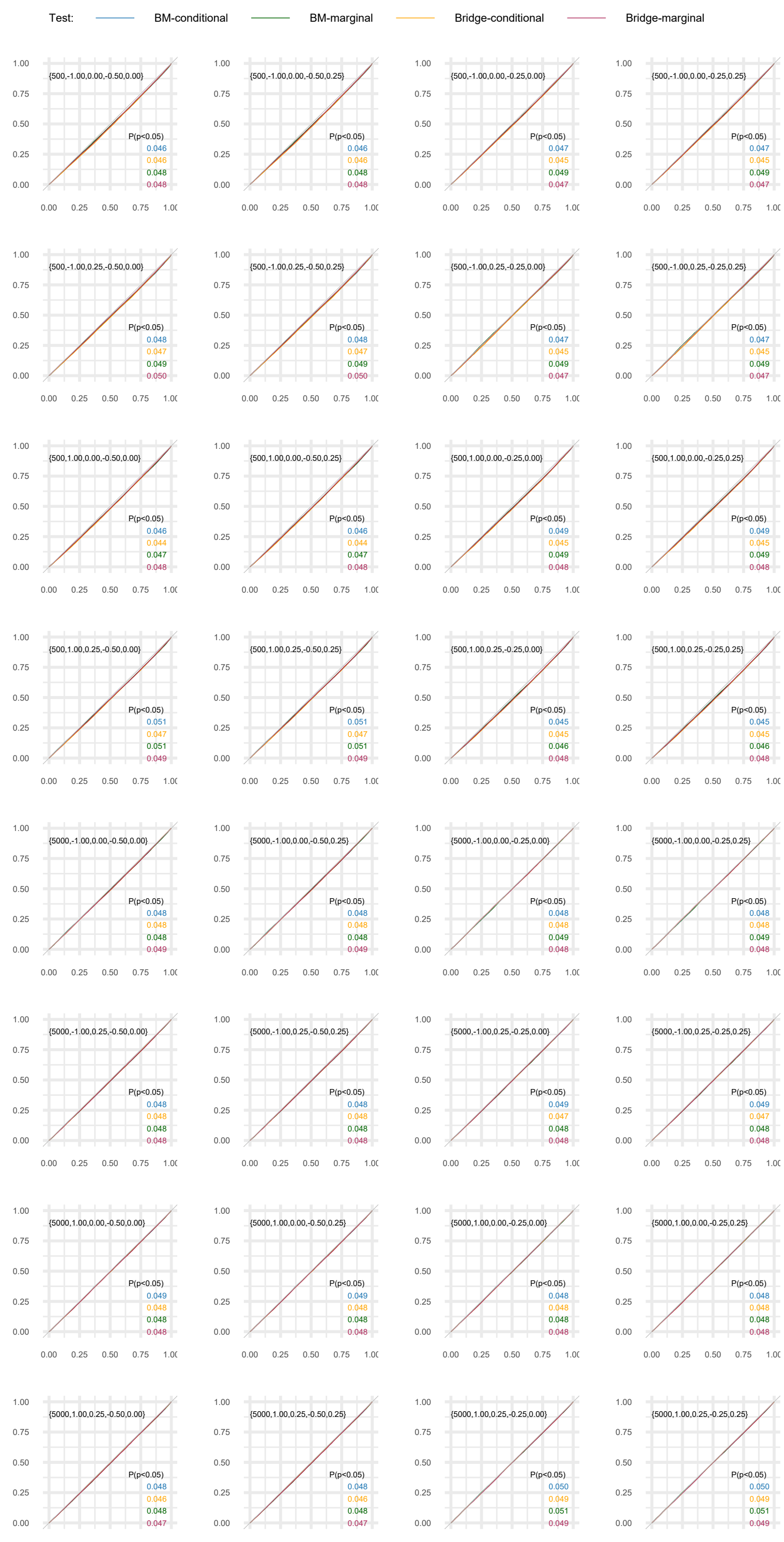

Sensitivity of the conditional approach to miscalibrated baseline risks

The purpose of these additional simulations is to demonstrate the dependence of the conditional test (Approach 1) on baseline risks being calibrated. In this simple setup, we start with the default logistic model we used in the main simulations (main text):

$$\text{logit}(P(Y = 1)) = 0 + 0.25x - 0.5a + 0.25xa,$$

with $x \sim Normal(0, 1)$ being the sole predictor. We generated validation samples of different sizes from this setup. The predicted ITE remains as the true value of the difference between risk for the untreated and treated, respectively.

Once predicted ITEs are obtained, we modified the baseline predicted risk (π_0, the predicted risk in the untreated), using the following two-parameter transformation applied to the true baseline risk (p_0 - obtained by setting $a = 0$):

$$\text{logit}(\pi_0) = \beta_0 + \beta_1 \text{logit}(p_0)).$$

We used the following values in a factorial design: N={500,5000}, β_0={-1/2,0,1/2}, and β_1={2/3,1,3/2}. Each simulation was run for 10,000 iterations.

As we alter π_0 after deriving the predicted ITEs, this modification means predicted ITEs will remain calibrated. The marginal approach, which does not rely on predicted risks, is not affected (and hence is not studied here).

Results are provided in the Figure below. For both coefficients, both one-part and two-part (bridge) tests are anti-conservative (resulting in increased type I error rate) at any significance level. This is generally expected as the bias in the predicted baseline risk is expected to result in increments with expectations that deviate systematically from their null values.

Overall, these simulations generally represent pronounced miscalibration scenarios (in order to demonstrate the effect). The distortion in the type I error can be modest if the miscalibration of risks is more subtle - as is often the case when the calibration of the model on the risk domain is first ensured before the ITE calibration is considered.

Figure 2: Empirical CDF of p-values under H_0 but with potential miscalibration of predicted risks[1]

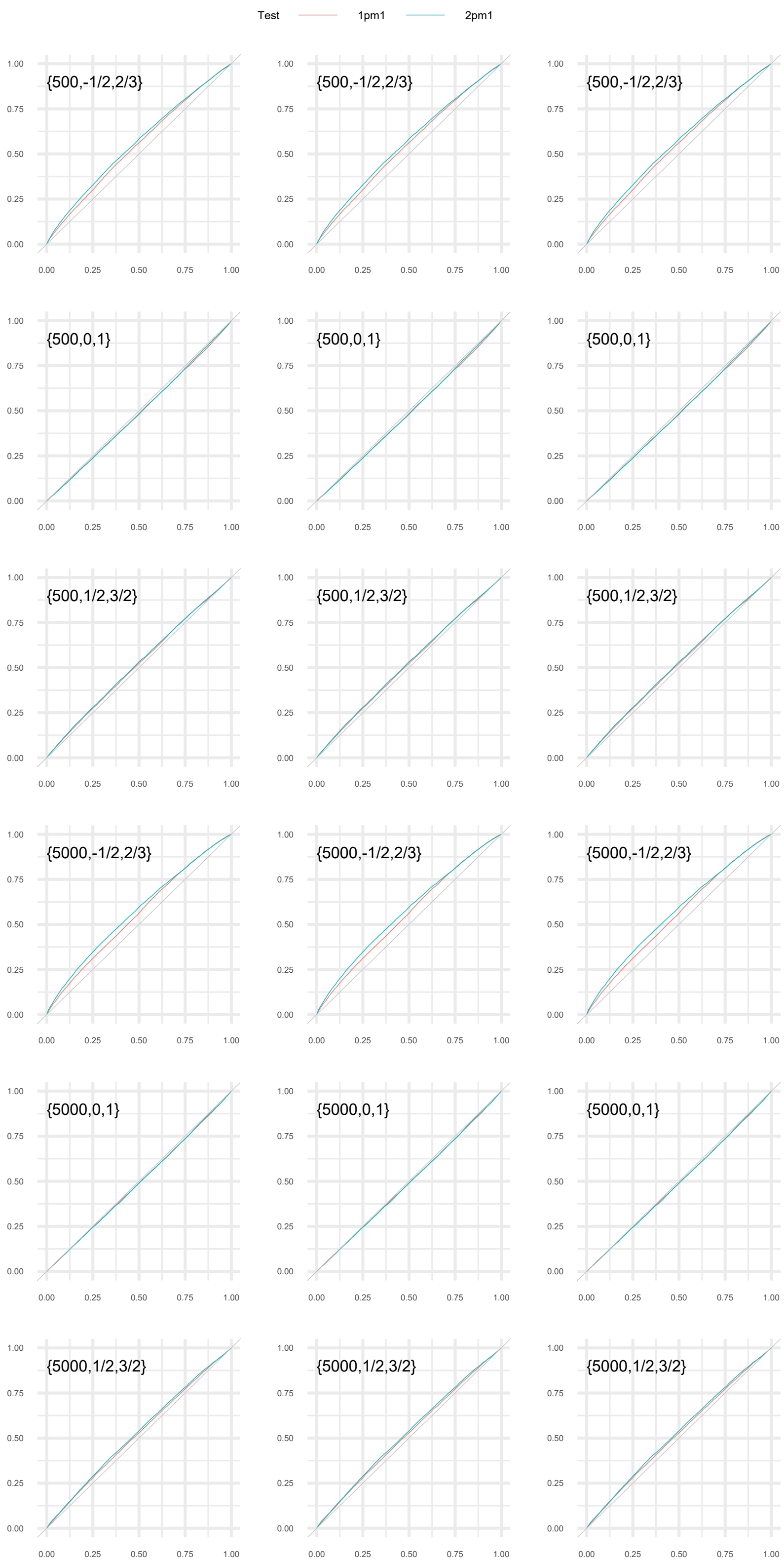

[1]Numbers in curly brackets are N, β_0, and β_1, in the same order

Section 2: Exemplary R code

This code uses the cumulcalib R package and the GUSTO data as an example of the implementation of the proposed tests. To illustrate a model with clearly detectable miscalibration, we deliberately develop the ITE model on a small random subset (one tenth) of the development data (an over-fitted model, mirroring the small-development-sample model of the main-text case study) and validate it on a one-tenth random subset of the validation sample.

The GUSTO data are available from the *predtools* package (on CRAN). The official version of the *cumulcalib* package can also be installed from CRAN. The output below was generated with version 0.1.0 of the package, archived under the tag `v0.1.0-SiMBenefit` on github.

```
###Un-comment and run the lines below to install the exact version of the
###cumulcalib package used for this paper (tag v0.1.0-SiMBenefit)
#library(remotes)
#remotes::install_github("resplab/cumulcalib@v0.1.0-SiMBenefit", force=TRUE)

library(predtools) #For GUSTO data
data("gusto")
library(cumulcalib)

df <- gusto[-which(gusto$tx=="SK+tPA"),] # Removing the double therapy arm
df$a <- ifelse(df$tx=="tPA", 1, 0)*1 # 1 for TPA 0 for other (SKA or SKA combo)
df$Y <- df$day30
df$kill <- (as.numeric(df$Killip)>1)*1 ## Killip Class Binary (0 = KC 1)

# Development sample: non-US; validation sample: US (as in the case study)
data_dev <- df[!df$regl %in% c(1, 7, 9, 10, 11, 12, 14, 15),] # Non-US data
data_val <- df[df$regl %in% c(1, 7, 9, 10, 11, 12, 14, 15),]  # US data

# Deliberately develop on a small (over-fitted) subset and validate on a subset,
# to obtain an ITE model with clearly detectable miscalibration
set.seed(122)
df_dev <- data_dev[sample(nrow(data_dev), round(nrow(data_dev) / 10)), ]
df_val <- data_val[sample(nrow(data_val), round(nrow(data_val) / 10)), ]

ite_model <- glm(Y ~ sex + age + miloc + pmi + kill + pmin(sysbp,100) + pulse +
                   a + a:sex + a:age, data = df_dev,
                  family=binomial(link="logit"))

#Creating counterfactual predicitons and predicted ite (h)
df_ <- df_val
df_$a <- 0
p0 <- predict(ite_model, newdata=df_, type="response")
df_$a <- 1
p1 <- predict(ite_model, newdata=df_, type="response")

df_val$p0 <- p0
df_val$h <- p0-p1
```

```
#Approach 1, Brownian motion test
res1 <- cumulcalibITE(df_val$Y, df_val$h, df_val$a, df_val$p0, method = "BM")
summary(res1)
```

```
## Moderate calibration assessment of individualized treatment effects
```

```
## Approach: conditional
## C_n (mean calibration error): 0.0363985965193455
## C* (maximum cumulative calibration error): 0.0488039015226009  (observed benefit > predicted)
##   Location of maximum cumulative error: time = 0.657662473334137, predicted ITE = -0.00331168848044092
##   Shape: the cumulative error reverses around predicted ITE = -0.00331 (observed benefit exceeds predicted below this point;
## Method: One-part Brownian motion (BM)
## S* (test statistic for cumulative calibration error): 3.91889501088661
## p-value: 0.000177911699480449
```

```r
# lines="vh" draws the vertical test-statistic line AND the horizontal
# significance threshold; the package default ("v") omits the threshold
plot(res1, stats_config = list(lines = "vh"))
```

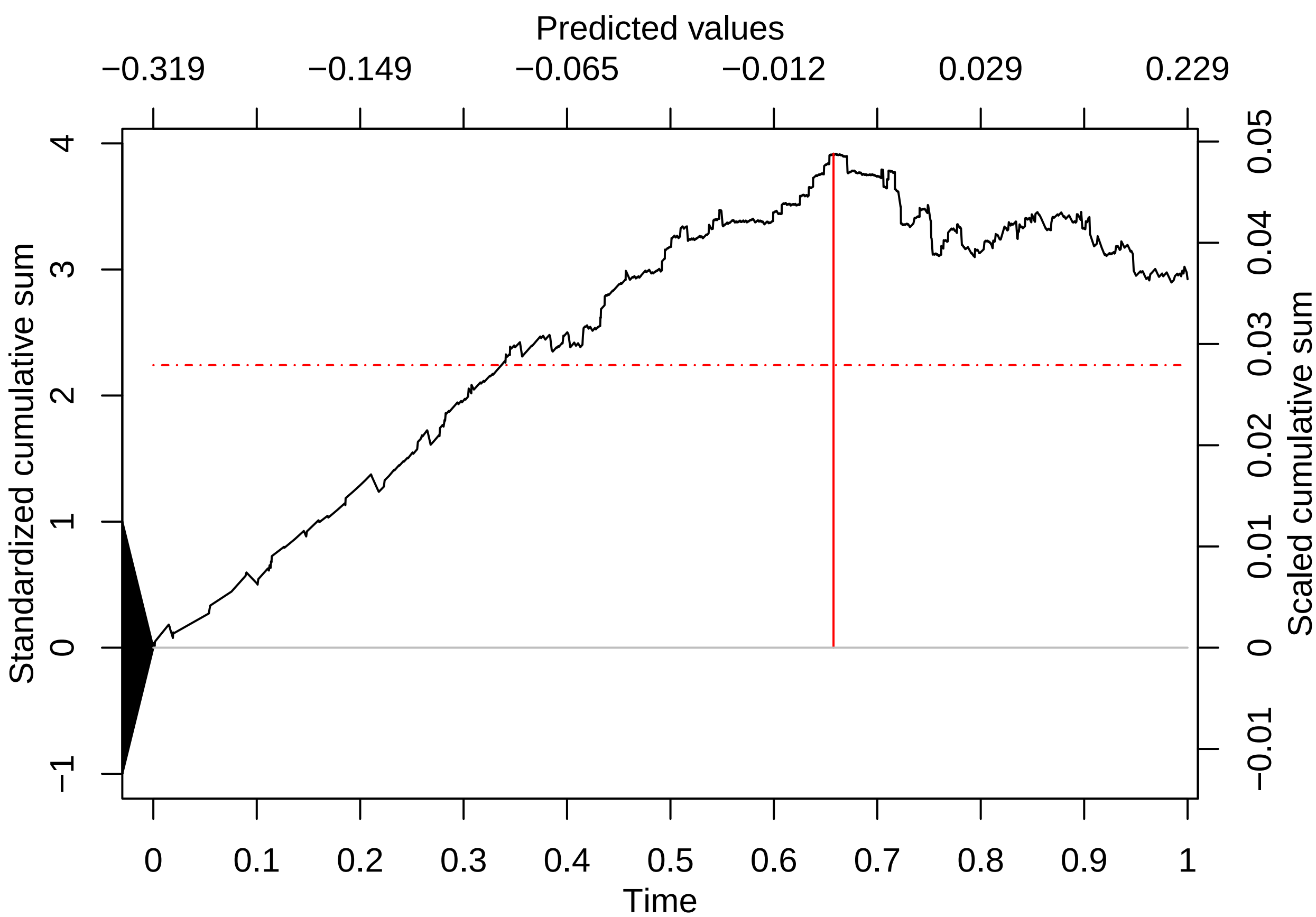


```r
#Approach 2, Brownian bridge test
res2 <- cumulcalibITE(df_val$Y, df_val$h, df_val$a, df_val$p0, method = "BB")
summary(res2)
```

```
## Moderate calibration assessment of individualized treatment effects
## Approach: conditional
## C_n (mean calibration error): 0.0363985965193455
## C* (maximum cumulative calibration error): 0.0488039015226009  (observed benefit > predicted)
##   Location of maximum cumulative error: time = 0.657662473334137, predicted ITE = -0.00331168848044092
##   Shape: the cumulative error reverses around predicted ITE = -0.00331 (observed benefit exceeds predicted below this point;
## Method: Two-part Brownian bridge (BB)
## S_n (Z score for mean calibration error): 2.92276383347919
## B* (test statistic for maximum absolute bridged calibration error): 1.99735740841779
## Component-wise p-values: mean calibration=0.00346939553914561 | Distance (bridged)=0.000685250527216574
## Combined p-value (Fisher's method): 3.3163615011933e-05
```

```r
plot(res2, stats_config = list(lines = "vh"))
```

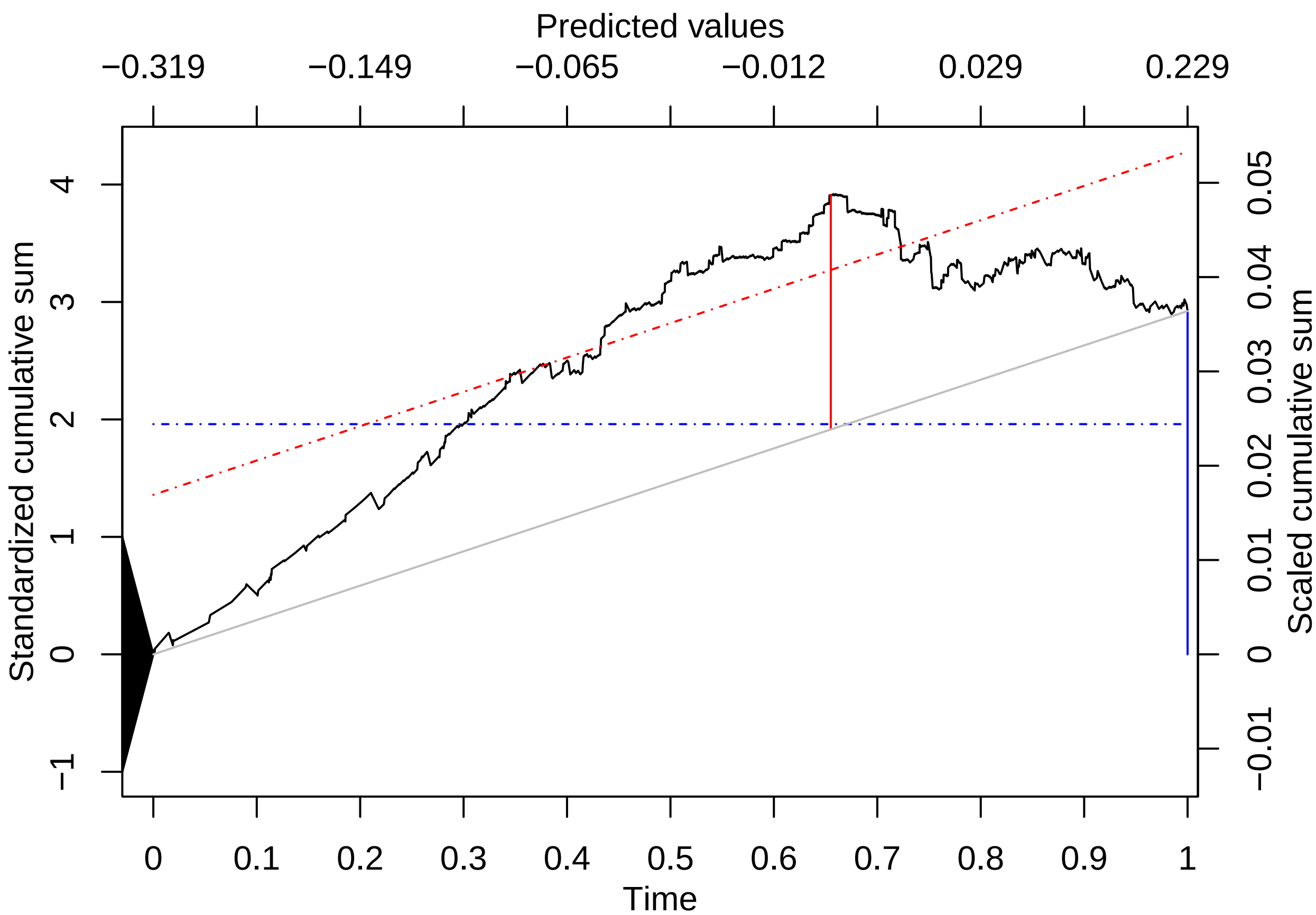

Section 3: Code and data availability

- The core functions are under the *cumulcalib* R package, available from CRAN (https://CRAN.R-project.org/package=cumulcalib) or GitHub (https://github.com/resplab/cumulcalib).
- The main function for individualized treatment effects is cumulcalibITE().
  - All results reported in this paper were generated with version 0.1.0 of the package, archived under the tag `v0.1.0-SiMBenefit` (commit `8af22ef`). This exact version can be installed via `remotes::install_github("resplab/cumulcalib@v0.1.0-SiMBenefit")`.
- The code generating the results in the paper is available from `https://github.com/resplab/papercode/tree/main/cumulcalibbenefit`.
  - The main file is `cumulcalibB_resgen.Rmd`.
  - Save this and `core.R` files in the same folder locally and make it the working directory in R.
  - The code also depends on other packages (eg *ggplot2*) for processing the results.
  - Please make sure the `res_path` folder in the first code chunk points to a folder on your local system.
